# Designing novel cabozantinib analogues as p-glycoprotein inhibitors to target cancer cell resistance using molecular docking study, ADMET screening, bioisosteric approach, and molecular dynamics simulations

**DOI:** 10.3389/fchem.2025.1543075

**Published:** 2025-02-27

**Authors:** Gajendra Singh Thakur, Ajay Kumar Gupta, Dipti Pal, Yogesh Vaishnav, Neeraj Kumar, Sivakumar Annadurai, Sanmati Kumar Jain

**Affiliations:** ^1^ Drug Discovery and Research Laboratory, Department of Pharmacy, Guru Ghasidas Vishwavidyalaya (A Central University), Bilaspur, Chhattisgarh, India; ^2^ Department of Pharmaceutical Chemistry, Bhupal Nobles’ College of Pharmacy, Udaipur, Rajasthan, India; ^3^ Department of Pharmacognosy, College of Pharmacy, King Khalid University, Abha, Saudi Arabia

**Keywords:** anti-cancer agent, newer analogues, bioisosteric approach, cabozantinib, molecular docking, MD simulation

## Abstract

**Introduction:**

One of the foremost contributors to mortality worldwide is cancer. Chemotherapy remains the principal strategy for cancer treatment. A significant factor leading to the failure of cancer chemotherapy is the phenomenon of multidrug resistance (MDR) in cancer cells. The primary instigator of MDR is the over expression of P-glycoprotein (P-gp), a protein that imparts resistance and facilitates the ATP-dependent efflux of various anticancer agents. Numerous efforts have been made to inhibit P-gp function with the aim of restoring the effectiveness of chemotherapy due to its broad specificity. The main objective has been to create compounds that either serve as direct P-gp inhibitors or interact with cancer therapies to modulate transport. Despite substantial in vitro achievements, there are currently no approved drugs available that can effectively “block” P-gp mediated resistance. Cabozantinib (CBZ), a multi-kinase inhibitor, is utilized in the treatment of various carcinomas. CBZ has been shown to inhibit P-gp efflux activity, thereby reversing P-gp mediated MDR. Consequently, P-gp has emerged as a critical target for research in anti-cancer therapies.

**Methods:**

The purpose of this study was to computationally identify new andsafer analogues of CBZ using bioisosteric approach, focusing on improved pharmacokinetic properties andreduced toxicity. The physicochemical, medicinal, and ADMET profiles of generated analogues were computed using the ADMETLab 3.0 server. We also predicted the drug likeness (DL) and drug score (DS) of analogues. The molecular docking studies of screened analogues against the protein (PDB ID: 3G5U) were conducted using AutoDock Vina flowing by BIOVIA Discovery Studio for visualizing interactions.Molecular dynamics (MD) simulation of docked ligands was done using Schrödinger suite.

**Results and Discussion:**

The docking scores for the ligands CBZ01, CBZ06, CBZ11, CBZ13, CBZ25, CBZ34, and CBZ38 ranged from −8.0 to −6.4 kcal/mol against the protein (PDB ID: 3G5U). A molecular dynamics (MD) simulation of CBZ01, CBZ13, and CBZ38 was conducted using the Schrödinger suite, revealing that these complexesmaintained stability throughout the 100 ns simulation.

**Conclusion:**

An integrated computational approach combining bioisosteric approach, molecular docking, drug likeness calculations, and MD simulations highlights the promise of ligands CBZ01 and CBZ13 as candidates for the development of potential anticancer agents for the treatment of various cancers.

## 1 Introduction

Cancer continues to be a primary cause of worldwide mortality. The International Agency for Research on Cancer (IARC), a division of the World Health Organization (WHO), which is dedicated to cancer studies, has published its recent estimates regarding the global cancer burden. An estimated 20 million new cancer cases and over 9.7 million cancer-related deaths occurred in 2022. Around 53.5 million people survived for at least 5 years after their diagnosis. Lung cancer has emerged as the most prevalent cancer globally, with 2.5 million new cases, representing 12.4% of all new diagnoses. Following lung cancer, female breast cancer ranked second in prevalence at 11.6%, succeeded by colorectal cancer (9.6%), prostate cancer (7.3%), and stomach cancer (4.9%). Forecasts show that the number of new cancer cases could escalate to 35 million by 2050, which marks an increase of 77% compared to the figures recorded in 2022. This significant rise in the global cancer burden is largely attributed to an aging population, overall population growth, and shifts in exposure to various risk factors, many of which are linked to socioeconomic development. Key contributors to the rising incidence of cancer include tobacco use, alcohol consumption, and obesity, while air pollution remains a significant environmental risk factor (Ferlay et al., 2024; Sung et al., 2021).

The treatment of cancer is recognized as a particularly challenging endeavour, encompassing methods such as chemotherapy, radiotherapy, and surgical interventions (Cao et al., 2024; Bray et al., 2024; Liu B. et al., 2024). A significant obstacle in contemporary cancer research is the emergence of drug resistance and the recurrence of cancer, which often undermine the effectiveness of even the most potent anti-cancer therapies (Anand et al., 2023; Khan et al., 2024). In the context of chemotherapy, one of the primary reasons for treatment failure is the phenomenon of multidrug resistance (MDR) observed in cancer cells. The over expression of P-glycoprotein (P-gp), an ATP-binding cassette transporter, is a key factor contributing to MDR, as it enhances the efflux of various anti-cancer agents from the cells. P-gp was the first protein identified to be linked to drug resistance (Bukowski et al., 2020; Lin et al., 2016a; Lin et al., 2016b; Goebel et al., 2021; Zhang et al., 2022; Garrigues et al., 2002). P-gp, identified in 1976, is a membrane glycoprotein with an estimated molecular weight of 170 kDa, found in drug-resistant Chinese hamster ovary cells. It consists of two transmembrane domains (TMDs) and two nucleotide-binding domains (NBDs) (Kim and Chen, 2018; Tian et al., 2023; Sun et al., 2023). The NBDs, located in the cytoplasm, facilitate the movement of substrates by transferring energy across cellular membranes, while the TMDs, composed of six transmembrane helices, provide substrate selectivity (Vasiliou et al., 2009; Johnson and Chen, 2018).

P-gp is recognized as a significant MDR transporter, particularly in relation to its role in conferring resistance to cancer chemotherapy. Its expression has been found to be elevated in various tumor types, such as osteosarcoma, kidney cancer, liver cancer, breast cancer, gastric cancer, lung cancer, and colorectal cancer, which contributes to the development of chemotherapy resistance (Karthika et al., 2022; Heming et al., 2022; Sharom, 2011). Cabozantinib (CBZ), a small-molecule, multitargeted tyrosine kinase inhibitor, is utilized in the treatment of several cancers, including metastatic medullary thyroid cancer, RCC, and HCC. Patients undergoing CBZ therapy may experience a range of toxicities, including hepatotoxicity and renal impairment, which can be severe or potentially life-threatening (Srigadha et al., 2023; Markowitz and Fancher, 2018; Choueiri et al., 2015). According to LiverTox, the hepatotoxicity likelihood score of cabozantinib is E*, unproven but probably rare cause of clinically apparent liver damage (National Center for Biotechnology Information, 2025). According to DrugBank, cabozantinib has extensive plasma protein binding (≥99.7%) (National Center for Biotechnology Information, 2025). The toxicities of cabozantinib may affect the patient’s quality of life. The most common adverse events (AEs) are diarrhea, fatigue, hypertension, hand-foot syndrome, weight loss, nausea, stomatitis, gastrointestinal perforation, hypothyroidism and myelotoxicity (Schmidinger and Danesi, 2018). Adverse reactions were recorded from clinic reports and the most common were hypertension, mucositis/hand-foot skin reaction (HFSR), or gastrointestinal toxicity (Martini et al., 2022). Cabozantinib has a higher risk of hepatotoxicity (Wang K. et al., 2024). Krens et al. (2022) reported that cabozantinib is registered at a fixed dose of 60 mg. However, 46%–62% of patients in pivotal studies required dose reduction due to toxicity. Consequently, it is crucial to modify the structure of the CBZ molecule to develop analogues that are less toxic and safer. Adverse effects associated with CBZ treatment include diarrhea, hypertension, hand-foot syndrome, weight loss, reduced appetite, stomatitis, and nausea (Schwartz et al., 2020; Rimassa et al., 2019; Zuo et al., 2015). Numerous reports have documented hepatotoxicity and a variety of dose-dependent side effects linked to CBZ (Barnhill et al., 2020; Andrade et al., 2019; Chiruvella et al., 2020). Due to these toxicities, it is imperative to modify the structure of the CBZ molecule to create safer and less toxic analogues.

The development of a lead chemical into a pharmaceutical agent presents significant challenges and often incurs high costs. Most candidates fail primarily due to pharmacokinetic and metabolic complications rather than a lack of efficacy. Even when a lead molecule exhibits the desired pharmacological effect, it may still present adverse side effects, characteristics that hinder its bioavailability, or chemical structures that impede its metabolism and elimination from the body. To address these issues, researchers employ the strategy of bioisosterism, which involves the selective modification of lead compounds to create safer and more effective medications. Bioisosterism is often perceived as a qualitative and intuitive concept. The common physicochemical properties of a set of bioisosteres are believed to contribute to their capacity to elicit similar biological responses. By leveraging an understanding of pharmacophores and physicochemical features, researchers are increasingly substituting bioisosteres for functional groups, thereby enhancing the potential for the development of innovative therapeutic agents. The foundational work of Langmuir in 1919 laid the groundwork for the bioisosterism approach to modifying lead compounds. Through the bioisosteric method, chemists can adjust various characteristics of the lead compound, including its size, shape, electrical distribution, polarizability, dipole moment, polarity, lipophilicity, and pKa, while ensuring effective binding to the target. Consequently, the strategic application of the bioisosteric method allows for the modification of lead compounds to yield more favorable therapeutic drugs with enhanced potency, selectivity, improved physical and metabolic properties, and minimized side effects (Giordano et al., 2022; Das et al., 2022; Jayashree et al., 2022).

**FIGURE 1:**
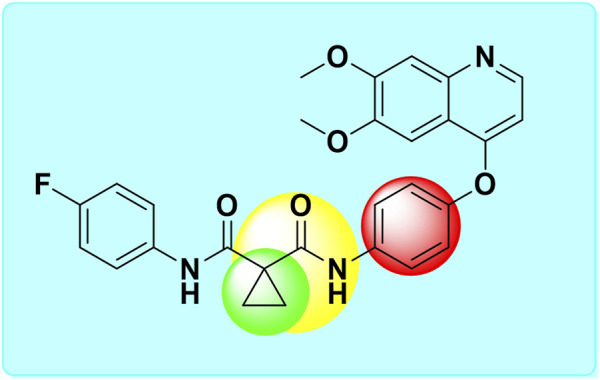
Structure of cabozantinib and its bioisosterically modified groups [Cyclopropyl (green circle), amide cyclopropyl (yellow circle), and phenyl (red circle)].

**FIGURE 2:**
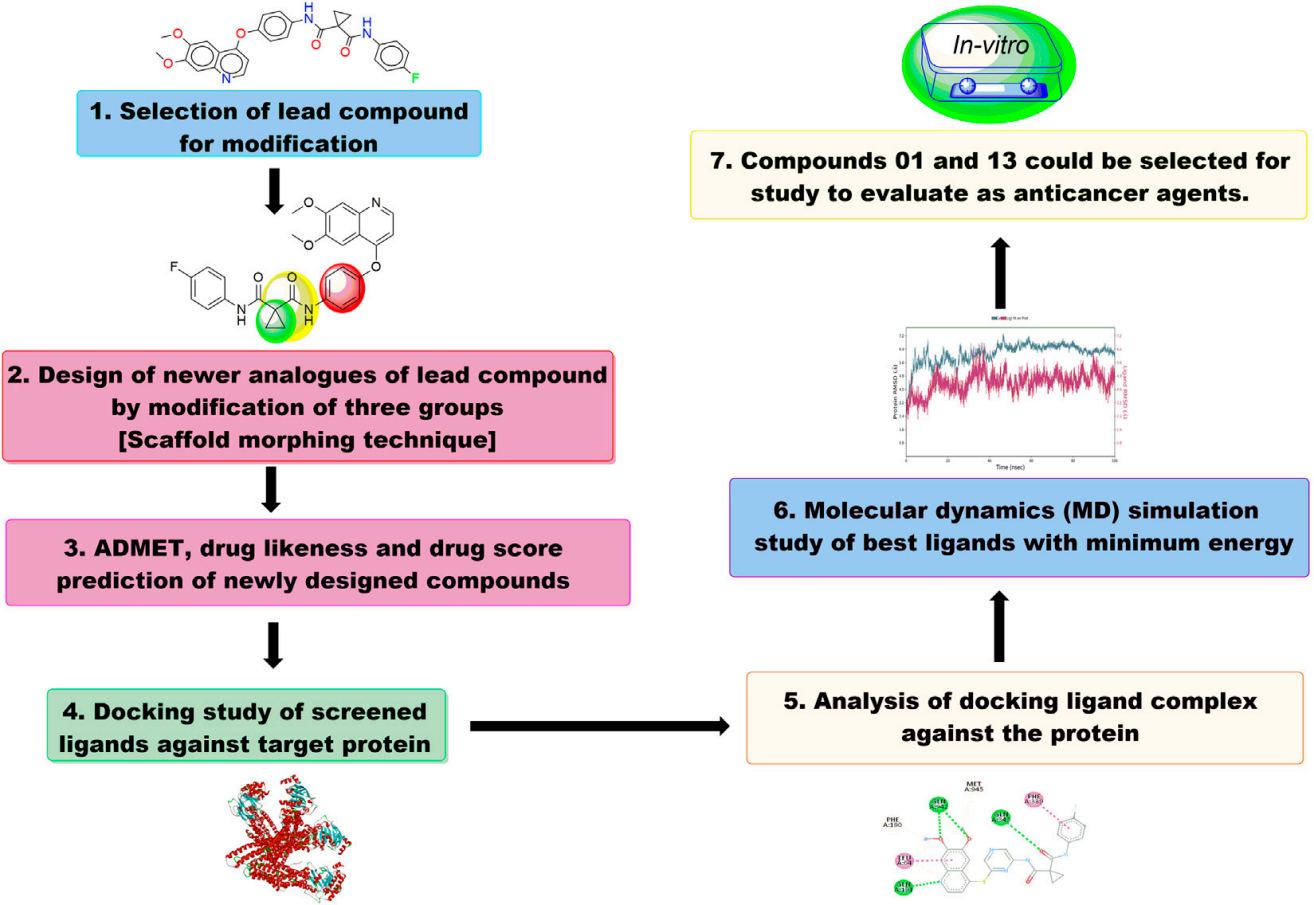
The overall work flow of the present study.

Structure-based drug design represents a highly effective and powerful approach within the broader context of drug discovery. The drug development process, which encompasses combinatorial chemistry, various screening methodologies, and the assessment of parameters such as absorption, distribution, metabolism, excretion, and toxicity (ADMET), can be significantly accelerated through the use of computational resources (Anderson, 2003; Jiang et al., 2018; Nie et al., 2020; Ejalonibu et al., 2021; Gao et al., 2023; Li et al., 2024). Recently, molecular docking has become an essential element of *in silico* drug discovery. This technique focuses on predicting the atomic-level interactions between proteins and small molecules. The accessibility of free software for conducting docking simulations of protein-ligand systems has facilitated a growing number of studies utilizing this approach, with tools like AutoDock, ArgusLab, and GOLD providing docking estimates for a variety of receptor-ligand interactions. The docking interactions suggest the most favorable docked conformers based on the overall energy of the system. Additionally, it assists in identifying the specific amino acids of the protein that interact with the test molecules, thereby helping to evaluate the affinity of the tested molecule for the target protein (Bhagat et al., 2021; Du et al., 2016; Ferreira et al., 2015; Duan et al., 2024; Kang et al., 2018). To elucidate the molecular basis of protein function, molecular dynamics (MD) simulation is the predominant computational method employed to investigate the structure and dynamic behavior of proteins. Based on the docking scores and interactions, we selected three complexes of CBZ analogues for MD simulations (Salo-Ahen et al., 2020; Gao et al., 2019; Naresh and Guruprasad, 2020; Ajmal et al., 2016). The primary objective of this study is to modify various groups within the CBZ molecule, specifically phenyl, amide cyclopropyl, and cyclopropyl groups (Figure 1). The goal is to create CBZ analogues that are both safer and more effective. Additionally, we conducted ADMET predictions, molecular docking analyses, and MD simulations on the chosen CBZ analogues. The overall workflow of the present study is shown in Figure 2.

## 2 Materials and methods

### 2.1 Designing of CBZ Bioisosteres

The smile notation for CBZ analogues was acquired from DrugBank, a prominent chemical information platform. The bioisosteres of CBZ were created utilizing the MolOpt software, an online tool that produces bioisosteres through data mining, similarity assessments, and AI generative models (Shan and Ji, 2020; Subbaiah and Meanwell, 2021).

### 2.2 Pharmacokinetic and toxicological (ADMET) Profile Predictions

The pharmacokinetic and toxicological profile were forecasted utilizing the ADMETLab 3.0 online software. This comprises 119 quantitative and qualitative predictable endpoints, which effectively and thoroughly assess ADMET characteristics for novel ligands that exhibit ADMET properties similar to those observed in mammals (Dulsat et al., 2023; Gupta et al., 2024; Lou et al., 2023; Liu M. et al., 2024).

### 2.3 Drug likeness (DL) and drug score (DS) prediction

The primary variables leading to the failure of drug candidates in clinical trials are often intolerable toxicity levels or unfavourable pharmacokinetic characteristics. Therefore, it is crucial to conduct evaluations of DL and DS in the early stages of the drug development process. The calculations for drug score and drug likeness were performed utilizing the Osiris property explorer (Sun et al., 2022).

### 2.4 Molecular docking studies

Molecular docking is essential in drug discovery and structural molecular biology for predicting the main binding mode(s) of a ligand with a protein of known 3D structure. In this context, commonly used docking-related terminology (such as Apo protein, positive control, native ligand, and co-crystal inhibitors) is employed to elucidate the core principles of molecular docking, which encompass binding affinity, binding orientation, and ligand interactions. The docking analysis was conducted using AutoDock Vina (ADV) as the primary tool, and the interactions were evaluated with Discovery studio software (De Ruyck et al., 2016; Trott and Olson, 2010; Wang Z. et al., 2024; Ikwu et al., 2020). P-gp is integral to the process of cellular detoxification, as it facilitates the removal of a wide range of chemically diverse toxins. However, it is also linked to the phenomenon of multidrug resistance (MDR) in cancer treatments. Among the various transporters associated with MDR, P-gp is the most significant member of the ATP-binding cassette (ABC). P-gp demonstrates remarkable poly-specificity, enabling it to recognize a broad spectrum of compounds with molecular weights ranging from 330 Da to 4,000 Da. The X-ray crystallographic analysis of apo-P-gp with a resolution of 3.8 Å shows an internal cavity that is approximately 6,000 Å^3^, with a separation of 30 Å between the two nucleotide binding domains (NBD). In addition, two additional P-gp structures, which complex with cyclic peptide inhibitors, illustrate different drug binding sites within the inner cavity that have a stereoselectivity influenced by hydrophobic and aromatic interactions. Therefore, the P-gp protein, which has the ability to absorb a variety of substrates, is a defining feature of its function and makes a structural understanding of the poly-specific drug binding necessary for the rational development of anticancer agents and MDR inhibitors (PDB ID: 3G5U), chosen for the present study.

#### 2.4.1 Protein preparation

The crystal structure of the P-gp (PDB ID: 3G5U) was acquired from the Protein Data Bank (Aller et al., 2009). To prepare the protein for docking studies, we initially introduced hydrogen atoms, applied Kollman charges, and eliminated water molecules. Subsequently, we saved the modified structure in PDBQT format after addressing the missing atoms (Sastry et al., 2013).

#### 2.4.2 Ligand preparation

The ligands 2D chemical structures were created utilizing ChemDraw. Subsequently, the 2D representations of CBZ and their corresponding analogues were converted into 3D structures through Chem3D software. The newly developed CBZ bioisosteres underwent energy minimization in Chem3D and were subsequently saved in SDF format. Using OpenBabel, the ligands were transformed into MOL2 format (Zielesny, 2005). These ligands were then incorporated into ADV tool and saved in pdbqt format to facilitate the docking process. Additionally, protein was also dragged into the ADV tool, and a grid box was prepared that defines the boundary for the docking process.

#### 2.4.3 Protein–ligand interactions using ADV

Utilizing the ADV software, we conducted docking study involving the ligands and the protein. The entire active site of the protein was encompassed within a grid box with the size of dimensions at X = 84, Y = 84 and Z = 84. The center of the grid was positioned at X = 28, Y = 86, and Z = 40. The default configurations for other docking parameters, such as ADV settings, crossover rates, and gene mutation rates, were maintained. The interaction between ligand and amino acid residue of protein were studied using Discovery Studio to produce 2D and 3D pose of interactions. The binding site of the target protein includes LEU64, PHE724, GLN942, MET945, PHE974 and VAL978, which are used to determine potential binding sites of the target protein with respect to the designed ligands (Xiang et al., 2015).

### 2.5 Molecular dynamics (MD) simulation

The MD simulation aims to explore the dynamic behavior and stability of protein-ligand complexes. We selected three complexes based on their interaction profiles and docking scores. The simulations were executed on an Acer workstation operating with Ubuntu 22.04. The Desmond program, part of the Schrödinger suite, was utilized to perform the MD simulations and to assess the docking of molecules, evaluating the efficacy of the predicted ligands (Elekofehinti et al., 2021). The protein-ligand complexes were constructed using the ‘System Builder’ tool. Following a reduction in volume, we opted for the SPC water model configured in an orthorhombic arrangement. The periodic boundary conditions for the X, Y, and Z-axes of the protein-ligand complex were established at 10 × 10 × 10 Å. Additionally, the crystal structure of the P-gp (PDB ID: 3G5U) served as a reference, illustrating its ability to accommodate 50.447 mM sodium and 53.855 mM chloride ions. Ions and salts within a 20 Å radius were omitted from the neutralization simulation. Before commencing the MD simulations, we applied the OPLS 2005 force field to minimize the energy of the complex, facilitating its transition to an equilibrium state. The OPLS 2005 force field, known as Optimized Potentials for Liquid Simulations, is a well-known force field in molecular mechanics designed to effectively simulate molecular interactions, particularly in the context of small organic molecules and biomolecular systems. This version represents an advance over the original OPLS force field and provides greater precision for simulations (González, 2011; Banks et al., 2005). To refine the complexes, we employed a minimization approach based on the steepest descent method. The complexes were subsequently heated to 300 K, achieving equilibrium after 1000 steps, with a time step of 100 ns. The final production run for the complexes was carried out over a period of 100 ns.

#### 2.5.1 Binding free energy calculations

The binding affinity of ligand-protein complexes, represented by binding free energy, was assessed through the binding energies protocol available in the Desmond program. The complexes CBZ01-3G5U and CBZ13-3G5U, which were produced following molecular dynamics (MD) simulations, underwent analysis for binding energy estimation. Both Poisson-Boltzmann and generalized Born models, in conjunction with surface area continuum solvation methods (MM-PBSA and MM-GBSA), were utilized for solvation analysis. Furthermore, binding energy calculations were performed without considering solvation effects. The free energy derived from the MM-PBSA method was computed using the gmx_MMPBSA tool, which necessitates the input of “.top” and “.trr” files. To generate these files, the Desmond Composite Model System files (.cms) were initially converted using the InterMol software (https://github.com/shirtsgroup/InterMol), leading to the creation of “.gro” and “.top” files. Subsequently, the Desmond trajectory was imported into VMD and saved in the “.trr” format. After preparing all required input files, MM-GBSA calculations were carried out using the gmx_MMPBSA tool. The MM-GBSA approach integrates molecular dynamics simulations with thermodynamic principles, enabling the calculation of the total binding free energy between a ligand and a protein, as illustrated in Equation 1 (Wang et al., 2019; Bouricha and Hakmi, 2024; Matore et al., 2023).

$$∆G_{\text{binding}}=∆G_{\text{MM}}+∆G_{\text{sol}}-T∆G$$ (1)

## 3 Results and discussion

### 3.1 Bioisosteres of cabozantinib

Bioisosterism represents a strategy in medicinal chemistry that employs a lead compound as a primary method for molecular modification, aimed at the rational development of new pharmaceuticals (Karmacharya et al., 2021). We have applied the bioisosterism to improve ADMET profile and reduce undesirable toxic effects. MolOpt produced 592 analogues of CBZ, targeting various groups including phenyl, amide cyclopropyl, and cyclopropyl within the CBZ drug framework. The compounds that were screened are detailed in Supplementary Table S1.

### 3.2 Physicochemical properties prediction

The aim of molecular property prediction is to ascertain the physicochemical, bioactive, toxicological, and other characteristics of a target compound based on its molecular structure. Supplementary Table S1 presents the predicted molecular properties for CBZ bioisosteres. All analogues complies with Lipinski’s rule of five, suggesting that these drug candidates possess favorable absorption and bioavailability. Furthermore, all analogues demonstrated a commendable topological polar surface area (TPSA) score, highlighting their capability to permeate cell membranes and reach target sites in the body.

### 3.3 Medicinal properties prediction

In the initial stages of drug development, the selection of molecules based on their drug-likeness is of paramount importance. This concept encompasses eight characteristics that are indicative of drug-related properties. The quantitative estimation of drug-likeness (QED) scores for the designed analogues, including CBZ01-02, CBZ05, and CBZ23-CBZ24, fall below the desirable QED score threshold (>0.67) but exceed the score of a standard drug (0.31). In the realm of drug design, predicting synthetic accessibility is a vital task that entails evaluating the ease of laboratory synthesis for a specific molecule. The synthetic accessibility scores for all designed analogues were determined to be within an acceptable range (<6). A research team from the Medicinal Chemistry Department of *in silico* Medicine has introduced the original descriptor MCE-18, which outlines the essential features of “next-generation” molecules and examines the evolution of medicinal chemistry over time (Ivanenkov et al., 2019). We utilized MCE-2018 to evaluate the efficacy of newly designed molecules such as CBZ03-04, CBZ05, CBZ12-14, CBZ16, CBZ18, CBZ23-25, CBZ27-29, CBZ31, CBZ33, CBZ37, CBZ39, CBZ48, CBZ49-50, CBZ57, and CBZ63, which yielded scores exceeding 63. Consequently, these analogues require visual inspection to determine their drug-likeness and target profiles. With the exception of CBZ07, CBZ08, CBZ26, CBZ45, CBZ47, CBZ64-66 and CBZ80, all designed analogues met the acceptance criteria of Lipinski’s rule of five (Karami et al., 2022). Additionally, Pfizer’s rule was satisfied by all analogues, indicating favorable physicochemical properties with potential for cellular permeability. The GT rule found accepted for anlogues such as CBZ05-06, CBZ12, CBZ14-16, CBZ25, CBZ27-29, CBZ31, CBZ35-36, CBZ52-54, and CBZ59. The medicinal properties of CBZ analogues are presented in Table 1.

**TABLE 1:** Medicinal, drug likeness (DL) and drug score (DS) properties of CBZ analogues.

Compound no.	QED	Synth	MCE-18	Lipinski	Pfizer	GSK	GT	DL	DS
CBZ01	0.45	2.97	70	0	0	1	1	4.56	0.59
CBZ02	0.45	2.82	71	0	0	1	1	4.45	0.49
CBZ03	0.30	3.80	99	0	0	1	1	2.40	0.51
CBZ04	0.36	3.20	82	0	0	1	1	−3.09	0.22
CBZ05	0.46	3.28	99	0	0	1	0	3.68	0.52
CBZ06	0.36	2.91	64	0	0	1	0	4.14	0.21
CBZ07	0.35	3.02	66	1	0	1	1	3.44	0.24
CBZ08	0.31	3.04	66	1	0	1	1	3.45	0.23
CBZ09	0.35	2.98	66	0	0	1	1	3.52	0.19
CBZ10	0.34	2.85	66	0	0	1	1	2.83	0.41
CBZ11	0.26	3.12	57	0	0	1	1	0.85	0.44
CBZ12	0.32	3.23	96	0	0	1	0	2.53	0.46
CBZ13	0.43	3.66	102	0	0	1	1	3.76	0.45
CBZ14	0.33	3.33	82	0	0	1	0	3.85	0.53
CBZ15	0.35	2.83	57	0	0	1	0	2.73	0.56
CBZ16	0.22	3.40	83	0	0	1	0	0.89	0.29
CBZ17	0.34	2.90	61	0	0	1	1	−21.00	0.22
CBZ18	0.28	3.35	89	0	0	1	1	0.31	0.41
CBZ19	0.28	3.02	56	0	0	1	1	3.06	0.20
CBZ 20	0.33	2.89	65	0	0	1	1	2.70	0.37
CBZ21	0.36	2.91	54	0	0	1	1	−0.34	0.30
CBZ22	0.32	2.92	65	0	0	1	1	4.86	0.43
CBZ23	0.41	3.67	101	0	0	1	1	3.48	0.50
CBZ24	0.41	3.31	99	0	0	1	1	2.23	0.45
CBZ25	0.31	3.16	79	0	0	1	0	−0.37	0.41
CBZ26	0.33	2.98	64	1	0	1	1	1.11	0.15
CBZ27	0.35	3.14	79	0	0	1	0	−108.00	0.37
CBZ28	0.28	3.31	79	0	0	1	0	2.33	0.47
CBZ29	0.38	3.03	79	0	0	1	0	2.16	0.49
CBZ30	0.35	2.90	64	0	0	1	1	1.12	0.35
CBZ31	0.39	3.11	79	0	0	1	0	2.49	0.59
CBZ32	0.14	2.72	68	0	0	1	1	0.00	0.29
CBZ33	0.21	3.15	95	0	0	1	1	1.70	0.39
CBZ34	0.21	3.05	56	0	0	1	1	−2.47	0.13
CBZ35	0.24	2.85	54	0	0	1	0	3.19	0.54
CBZ36	0.31	2.58	55	0	0	1	0	−51.00	0.19
CBZ37	0.31	3.41	88	0	0	1	1	1.88	0.41
CBZ38	0.23	2.86	65	0	0	1	1	2.05	0.24
CBZ39	0.21	3.16	95	0	0	1	1	2.18	0.39
CBZ40	0.25	2.87	63	0	0	1	1	1.33	0.33
CBZ41	0.30	2.51	63	0	0	1	1	2.31	0.35
CBZ42	0.31	2.62	63	0	0	1	1	1.67	0.37
CBZ43	0.32	3.01	67	0	0	1	1	2.55	0.32
CBZ44	0.27	2.66	65	0	0	1	1	1.53	0.34
CBZ45	0.24	3.25	65	1	0	1	1	4.13	0.41
CBZ46	0.30	2.90	67	0	0	1	1	3.72	0.37
CBZ47	0.24	3.26	64	1	0	1	1	−2.47	0.06
CBZ48	0.33	3.10	83	0	0	1	1	0.19	0.45
CBZ49	0.19	3.44	89	0	0	1	1	−6.42	0.22
CBZ50	0.37	3.37	85	0	0	1	1	2.57	0.46
CBZ 51	0.16	3.09	57	0	0	1	1	2.61	0.39
CBZ52	0.32	2.70	48	0	0	1	0	1.88	0.35
CBZ53	0.26	2.94	48	0	0	1	0	3.10	0.37
CBZ54	0.32	2.84	48	0	0	1	0	1.40	0.41
CBZ55	0.33	2.56	26	0	0	1	1	2.11	0.21
CBZ56	0.30	2.33	27	0	0	1	1	6.20	0.42
CBZ57	0.33	3.11	95	0	0	1	1	5.23	0.33
CBZ58	0.33	2.94	90	0	0	1	1	4.57	0.29
CBZ59	0.38	2.43	24	0	0	1	0	2.66	0.21
CBZ60	0.22	3.13	107	0	0	1	1	4.64	0.23
CBZ61	0.34	2.64	26	0	0	1	1	0.76	0.19
CBZ62	0.27	2.99	91	0	0	1	1	1.85	0.09
CBZ63	0.27	2.99	91	0	0	1	1	−3.64	0.03
CBZ64	0.18	2.62	26	1	0	1	1	−4.51	0.05
CBZ65	0.19	3.02	52	1	0	1	1	−4.51	0.05
CBZ66	0.19	3.02	52	1	0	1	1	1.89	0.05
CBZ67	0.30	2.60	63	0	0	1	1	4.89	0.41
CBZ68	0.28	3.06	52	0	0	1	1	-	-
CBZ69	0.27	3.35	52	0	0	1	1	−5.82	0.10
CBZ70	0.22	2.80	48	0	0	1	1	1.40	0.41
CBZ71	0.32	2.84	48	0	0	1	1	3.10	0.37
CBZ72	0.26	2.94	48	0	0	1	1	3.64	0.42
CBZ73	0.11	3.12	48	0	0	1	1	−1.18	0.15
CBZ74	0.33	2.86	48	0	0	1	1	2.94	0.42
CBZ75	0.29	2.86	48	0	0	1	1	3.39	0.30
CBZ76	0.31	2.48	24	0	0	1	1	4.36	0.44
CBZ77	0.29	2.86	48	0	0	1	1	2.97	0.09
CBZ78	0.22	2.87	48	0	0	1	1	3.34	0.41
CBZ79	0.27	3.17	52	0	0	1	1	−32.00	0.14
CBZ80	0.17	3.14	54	1	0	1	1	3.53	0.03
CBZ81	0.28	3.07	54	0	0	1	1	2.21	0.20
CBZ	0.31	2.42	63	0	0	1	1	2.08	0.34

QED, a measure of drug-likeness based on the concept of desirability; Synth, synthetic accessibility score; Fsp3, number of sp3 hybridized carbons/total carbon count; MCE-18, medicinal chemistry evolution in 2018; GT, golden triangle; DL, drug likeness; DS, drug score.

### 3.4 Prediction of DS and DL score

DL and DS are qualitative metrics utilized in drug design to assess the “drug-like” characteristics of a molecule, particularly in relation to factors such as bioavailability. These metrics are extensively integrated into the early stages of lead and drug discovery (Bickerton et al., 2012). During the initial phases of drug development, it is essential to screen compounds based on their drug-likeness and DS. Accurately predicting a compound’s drug-likeness is vital, as it offers valuable insights that can enhance the likelihood of transforming lead compounds into viable drugs, necessitating various drug-like attributes (Lagu et al., 2022). The DL score of compounds can indicate their potential effectiveness and safety. The DS serves as a comprehensive metric that consolidates factors such as toxicity concerns, cLogP, logs, molecular weight, and drug-likeness into a singular value. Among the analogues, CBZ56-57 and CBZ69 exhibit higher DL scores, while CBZ01-02, CBZ22, CBZ45, CBZ67, and CBZ76 demonstrate scores ranging from 4 to 4.5, surpassing the standard drug CBZ, which has a score of 2.08. The DL and DS scores of analogues are tabulated in Table 1.

### 3.5 Pharmacokinetic profile

Pharmacokinetics plays a crucial role in the drug discovery process by guiding the optimization of a compound’s absorption, distribution, metabolism, and excretion (ADME) properties. The primary objective is to ensure that a lead compound achieves a concentration-time profile within the body that supports the desired efficacy and safety outcomes. By incorporating ADMET data into the drug design framework, researchers can improve a compound’s solubility, permeability, and stability, ultimately developing clinical drug candidates capable of maintaining therapeutic concentrations for the required duration while minimizing potential risks. The predictable Caco-2 permeability scores offer valuable insights into the ability of substances to traverse intestinal cell membranes, which is a critical aspect of oral drug absorption. The Caco-2 scores for compounds CBZ01, CBZ04, CBZ06-15, CBZ17, CBZ19-21, CBZ24-31, CBZ33, CBZ34, CBZ37-47, CBZ49-51, CBZ55, CBZ64-66, CBZ77, and CBZ80 ≤ −5.15 log cm/s, indicating effective transport across intestinal membranes and favorable permeability. Conversely, the Caco-2 scores for compounds CBZ02, CBZ03, CBZ05, CBZ16, CBZ18, CBZ22, CBZ23, CBZ32, CBZ35, CBZ36, CBZ48, CBZ52-54, CBZ56-63, CBZ67-76, CBZ78, and CBZ79 fall ≥ −5.15, suggesting potentially poor permeability. Madin-Darby Canine Kidney (MDCK) cells are acknowledged as a reliable *in vitro* model for evaluating permeability, providing critical insights into the absorption efficiency of chemical substances within the body. The MDCK permeability scores for all evaluated compounds exceed 2 × 10⁻⁶ cm/s, indicating excellent MDCK permeability. This finding suggests that these compounds are likely to exhibit favorable permeability characteristics, making them promising candidates for effective systemic absorption.

The results show that all compounds reach a praising human intestinal absorption score (HIA) between 0 and 0.3, which indicates the significant potential for effective absorption in the human gastrointestinal tract. This favorable absorption profile implies that these compounds are likely to have high oral bioavailability, which is essential for the maintenance of the therapeutic drug level in systemic circulation. In addition, none of the compounds are classified as poorly absorbed (HIA+ with a value of more than 0.7), which reduces the likelihood of bioavailability problems associated with inadequate intestinal absorption. In addition, the results show that all compounds from 0 to 1 have values, which reflects the different probabilities of penetrating the blood-brain barrier (BBB). The findings further indicate that all compounds demonstrate the varying probabilities of traversing the blood-brain barrier (BBB). Plasma protein binding (PPB) is crucial in influencing the absorption, distribution, and pharmacodynamics of pharmaceuticals. The degree to which drugs associate with plasma proteins affects the concentration of the unbound drug that is available for therapeutic action. Compounds such as CBZ02, CBZ03, CBZ05, CBZ08, CBZ14-16, CBZ18, CBZ23, CBZ26, CBZ27, CBZ28, CBZ35, CBZ36, CBZ48, and CBZ51 demonstrate PPB values of 90% or less, indicating strong binding properties that promote an optimal equilibrium between the concentration of free drug and its therapeutic efficacy. In addition, compounds like CBZ34, CBZ41, CBZ52, CBZ53, CBZ55-66, CBZ68, CBZ69, CBZ71-73, CBZ75, and CBZ77-81 exhibit PPB values exceeding 90%, similar to CBZ, which has a PPB of 97.74%. The volume of distribution at steady state (VDss) serves as a pharmacokinetic indicator of how extensively a drug is distributed in the body relative to its plasma concentration. The expected VDss is measured in L/kg, with an optimal range of 0.04–20 L/kg indicated. All compounds show a projected VDSS score that falls in the range from 0.04 to 20 L/kg, which indicates superior distribution properties, with the exception of CBZ04, CBZ26, CBZ40-42, CBZ47, CBZ48 and CBZ51.

The unbound fraction (Fu) in plasma is a significant pharmacokinetic parameter that influences a drug’s efficacy and distribution. It represents the proportion of a drug that remains unbound to serum proteins, enabling it to traverse cellular membranes and exert its pharmacological effects. The Fu values for compounds CBZ01-03, CBZ05-10, CBZ13-18, CBZ20, CBZ23-28, CBZ31-33, CBZ35-37, CBZ39, CBZ43, CBZ44, CBZ46-48, CBZ50, and CBZ51 are greater than 5%. These compounds exhibit favorable unbound drug fractions, suggesting their potential to effectively penetrate cellular membranes and reach their designated targets, thereby indicating their promise for further drug development. In contrast, other compounds, including CBZ, present Fu values below 5%. The results show that all compounds serve as substrates for CYP3A4, a key and prevailing enzyme within the CYP450 family, which is essential for the metabolism of phase I. CYP3A4 is crucial for the oxidative metabolism of numerous medicines and endogenous compounds that occur mainly in the liver and intestine.

Plasma clearance (CL) is an indicator of the body’s ability to eliminate a drug from the plasma. This parameter directly affects the overall drug exposure and is crucial for determining the appropriate dosage required to maintain a stable plasma concentration. The compounds CBZ01-13, CBZ15-23, CBZ26, CBZ30, CBZ32-34, CBZ36-49, CBZ51-53, CBZ55, CBZ57, CBZ59-61, CBZ63, CBZ69, CBZ71-74, and CBZ76-78 exhibit CL scores ranging from 0 to 5 mL/min/kg, indicating excellent clearance profiles. Their elimination rates are effectively regulated, ensuring consistent drug exposure and optimal dosing. The half-life (T_1/2_) serves as a measure of the interplay between clearance and volume of distribution. A precise evaluation of these two parameters provides comprehensive insight into the pharmacokinetics of drugs within the body. The T1/2 values for the compounds CBZ01-11, CBZ13, CBZ17, CBZ18, CBZ20, CBZ22, CBZ23, CBZ26, CBZ31-33, CBZ37-40, CBZ42, CBZ44-49, and CBZ51 exceed 0.903, indicating that these compounds are suitable for dosing that maintains therapeutic drug levels. In contrast, the remaining compounds exhibit T_1/2_ values below 0.903, similar to that of CBZ, which implies the need for multiple dosing strategies. The pharmacokinetic (ADME) profile of CBZ analogues is mentioned in Table 2.

**TABLE 2:** ADME properties of CBZ analogues.

Compound no.	Caco-2 (log cm/s)	MDCK	HIA	BBB	PPB (%)	VD (L/kg)	Fu (%)	CYP3A4	CL (mL/min/kg)	T_1/2_
CBZ01	−5.02	Ex	Ex	0.07	91.41	0.24	7.63	+	3.38	1.31
CBZ02	−5.37	Ex	Ex	0.07	88.71	0.55	9.96	+	3.04	1.16
CBZ03	−5.37	Ex	Ex	0.06	80.73	0.59	17.74	+	2.39	1.83
CBZ04	−4.89	Ex	Ex	0.06	97.25	0.00	2.34	+	2.77	1.10
CBZ05	−5.24	Ex	Ex	0.02	88.73	0.54	10.07	+	2.89	1.21
CBZ06	−4.54	Ex	Ex	0.09	92.98	0.35	5.95	+	3.23	0.97
CBZ07	−4.83	Ex	Ex	0.07	91.47	0.36	6.92	+	2.04	1.31
CBZ08	−5.03	Ex	Ex	0.02	89.34	0.26	9.31	+	2.44	1.33
CBZ09	−4.69	Ex	Ex	0.16	93.15	0.52	5.41	+	1.76	1.24
CBZ10	−4.65	Ex	Ex	0.16	93.13	0.46	6.15	+	1.42	1.36
CBZ11	−4.59	Ex	Ex	0.00	93.90	0.18	4.97	+	2.46	1.16
CBZ12	−4.67	Ex	Ex	0.02	96.21	0.29	3.23	+	4.44	0.79
CBZ13	−4.91	Ex	Ex	0.03	90.99	0.70	6.71	+	3.13	1.01
CBZ14	−4.91	Ex	Ex	0.24	89.50	0.39	8.44	+	5.65	0.73
CBZ15	−5.05	Ex	Ex	0.06	84.21	0.18	17.03	+	3.45	0.80
CBZ16	−5.31	Ex	Ex	0.37	72.70	0.50	27.61	+	4.98	0.82
CBZ17	−4.69	Ex	Ex	0.00	93.73	0.07	5.58	+	2.32	1.08
CBZ18	−5.33	Ex	Ex	0.02	85.00	0.75	12.27	-	4.04	1.02
CBZ19	−4.71	Ex	Ex	0.43	95.71	0.22	3.60	+	4.67	0.58
CBZ 20	−4.64	Ex	Ex	0.35	91.97	0.49	7.16	+	2.91	0.93
CBZ21	−4.74	Ex	Ex	0.02	95.72	0.37	3.53	+	4.36	0.73
CBZ22	−5.17	Ex	Ex	0.01	96.48	0.01	3.09	+	3.51	1.04
CBZ23	−5.17	Ex	Ex	0.17	88.35	0.26	10.31	+	3.57	1.12
CBZ24	−5.02	Ex	Ex	0.40	91.97	0.40	6.55	+	5.13	0.69
CBZ25	−4.74	Ex	Ex	0.07	91.33	0.16	8.21	+	5.83	0.73
CBZ26	−4.92	Ex	Ex	0.03	87.00	−0.16	11.49	+	3.33	0.94
CBZ27	−5.06	Ex	Ex	0.02	76.44	0.17	22.43	+	5.97	0.85
CBZ28	−5.13	Ex	Ex	0.14	80.25	0.41	17.90	+	5.73	0.80
CBZ29	−4.77	Ex	Ex	0.03	93.92	0.15	4.63	+	5.67	0.71
CBZ30	−4.65	Ex	Ex	0.15	94.84	0.56	4.40	+	4.74	0.77
CBZ31	−4.66	Ex	Ex	0.11	91.72	0.11	7.59	+	5.63	1.00
CBZ32	−5.26	Ex	Ex	0.06	94.53	0.36	5.27	+	1.01	1.79
CBZ33	−4.80	Ex	Ex	0.07	92.09	0.57	6.40	+	1.84	1.14
CBZ34	−4.62	Ex	Ex	0.20	97.56	0.21	2.17	+	4.14	0.71
CBZ35	−5.20	Ex	Ex	0.12	86.91	0.37	12.34	+	5.93	0.69
CBZ36	−5.39	Ex	Ex	0.00	85.12	0.26	13.80	+	4.61	0.73
CBZ37	−5.04	Ex	Ex	0.04	91.72	0.17	7.03	+	2.86	1.10
CBZ38	−4.68	Ex	Ex	0.65	94.52	0.58	4.56	+	2.61	0.97
CBZ39	−4.94	Ex	Ex	0.10	92.41	0.67	6.03	+	1.59	1.28
CBZ40	−4.62	Ex	Ex	0.04	95.30	−0.05	4.07	+	2.42	1.22
CBZ41	−4.93	Ex	Ex	0.08	97.88	0.00	1.92	+	4.24	0.76
CBZ42	−4.81	Ex	Ex	0.05	96.65	−0.06	3.03	+	3.24	1.01
CBZ43	−4.89	Ex	Ex	0.77	93.39	0.67	5.12	+	3.28	0.82
CBZ44	−5.04	Ex	Ex	0.19	92.78	0.04	6.05	+	3.48	0.95
CBZ45	−5.14	Ex	Ex	0.02	96.76	−0.02	3.11	+	4.48	1.06
CBZ46	−4.82	Ex	Ex	0.73	92.38	0.37	5.75	+	2.87	0.99
CBZ47	−4.74	Ex	Ex	0.70	93.98	−0.08	5.19	+	3.19	1.09
CBZ48	−5.58	Ex	Ex	0.00	85.52	−0.30	10.21	+	2.57	1.26
CBZ49	−4.92	Ex	Ex	0.01	97.26	0.39	2.34	+	2.35	1.38
CBZ50	−4.98	Ex	Ex	0.07	92.26	0.34	5.86	+	5.70	0.63
CBZ 51	−4.91	Ex	Ex	0.00	89.72	−0.61	7.26	+	0.85	1.68
CBZ52	−5.48	Ex	Ex	0.03	98.52	0.74	1.08	+	3.57	0.24
CBZ53	−5.36	Ex	Ex	0.11	98.17	0.66	1.46	+	3.14	0.20
CBZ54	−5.39	Ex	Ex	0.18	97.06	0.60	1.83	+	7.36	0.46
CBZ55	−5.03	Ex	Ex	0.01	99.24	0.33	0.52	+	3.93	0.21
CBZ56	−5.20	Ex	Ex	0.08	98.47	0.89	1.25	+	7.39	0.10
CBZ57	−5.34	Ex	Ex	0.02	97.99	0.48	1.31	+	2.36	0.16
CBZ58	−5.21	Ex	Ex	0.03	98.00	0.38	1.22	+	5.79	0.10
CBZ59	−5.30	Ex	Ex	0.04	97.88	0.48	2.58	+	3.08	0.20
CBZ60	−5.75	Ex	Ex	0.02	98.79	0.57	1.40	+	4.40	0.09
CBZ61	−5.33	Ex	Ex	0.01	98.83	0.28	1.10	+	2.56	0.16
CBZ62	−5.38	Ex	Ex	0.04	98.00	0.67	1.30	+	7.22	0.22
CBZ63	−5.33	Ex	Ex	0.11	98.96	0.53	2.54	+	1.93	0.12
CBZ64	−5.04	Ex	Ex	0.03	99.44	0.52	1.02	+	7.83	0.12
CBZ65	−5.04	Ex	Ex	0.03	99.44	0.52	1.02	+	7.83	0.12
CBZ66	−4.83	Ex	Ex	0.03	99.74	0.54	0.67	+	6.91	0.17
CBZ67	−5.42	Ex	Ex	0.44	95.38	2.31	2.29	+	10.00	0.27
CBZ68	−5.52	Ex	Ex	0.08	98.91	2.16	1.90	+	5.30	0.86
CBZ69	−5.44	Ex	Ex	0.03	98.83	0.63	1.12	+	2.94	0.17
CBZ70	−5.39	Ex	Ex	0.18	97.06	0.60	1.83	+	7.36	0.46
CBZ71	−5.36	Ex	Ex	0.11	98.17	0.66	1.46	+	3.14	0.20
CBZ72	−5.26	Ex	Ex	0.04	99.15	0.63	1.10	+	2.00	0.28
CBZ73	−5.35	Ex	Ex	0.05	98.04	0.46	1.65	+	4.85	0.22
CBZ74	−5.53	Ex	Ex	0.20	96.01	1.25	2.22	+	4.35	0.35
CBZ75	−5.31	Ex	Ex	0.02	98.65	0.49	0.99	+	7.00	0.18
CBZ76	−5.53	Ex	Ex	0.56	96.10	1.64	2.39	+	4.74	0.31
CBZ77	−5.12	Ex	Ex	0.03	99.18	0.54	0.91	+	3.94	0.28
CBZ78	−5.42	Ex	Ex	0.04	98.41	0.51	1.37	+	4.10	0.27
CBZ79	−5.43	Ex	Ex	0.01	98.53	0.59	1.25	+	10.15	0.24
CBZ80	−4.69	Ex	Ex	0.04	99.20	0.59	0.70	+	7.33	0.14
CBZ81	−5.49	Ex	Ex	0.03	98.72	0.52	1.21	+	10.97	0.24
CBZ	−5.26	EX	Ex	0.04	97.74	1.00	1.20	+	3.51	0.18

Caco-2: the human colon adenocarcinoma cell lines; MDCK: madin-darby canine kidney cells; HIA: human intestinal absorption; PPB: plasma protein binding; BBB: blood–brain barrier; VD: volume distribution; Fu: the fraction unbound in plasms; Ex: excellent; (−): indicates inhibitor; (+): indicates substrate of human cytochrome P450 (five isozymes-1A2, 3A4, 2C9, 2C19 and 2D6); CL: the clearance of a drug; T_1/2_: the half-life of a drug.

### 3.6 Prediction of toxicity characteristics

In the initial stages of drug development, it is essential to precisely predict the ADMET properties to identify molecules that exhibit optimal pharmacokinetics while minimizing toxicity. The results indicated that all analogues exhibited lower human hepatotoxicity (H-HT) and drug-induced liver injury (DILI), compared to the reference drug (CBZ), suggesting a reduced risk of toxicity. The hERG (cardiotoxicity) score of all compounds, such as CBZ07, CBZ09, CBZ10, CBZ14, CBZ15, CBZ17, CBZ19, CBZ25-27, CBZ29, CBZ34, CBZ36, CBZ40, CBZ43, CBZ45-48, CBZ50, CBZ51, CBZ57, CBZ63-65, CBZ69 and CBZ72 show less than 0.3, indicating a lower risk of cardiotoxicity compared to the standard drug (0.604). Researchers have advocated for the use of mutagenicity data as a relatively quick and cost-effective method to assess long-term human health risks, including cancer in somatic cells and heritable mutations in germ cells. Given the strong correlation between mutagenicity and carcinogenicity, this assay is frequently employed in evaluating the mutagenic potential of compounds. The mutagenicity scores for certain analogues, including CBZ02, CBZ05, CBZ12-14, CBZ18, CBZ21, CBZ23-24, CBZ28, CBZ30, CBZ34, CBZ48, CBZ49, CBZ56-57, CBZ59-61, CBZ63, and CBZ79-CBZ81, fall within a safer range (from 0 to 0.3), indicating their suitability for non-mutagenic effects. Assessing acute toxicity in mammals, such as rats or mice, is a critical component of the safety evaluation for drug candidates, with toxicity testing conducted to identify potential adverse reactions. The ROA scores (ranging from 0 to 0.3) for certain analogues, including CBZ53, CBZ55-58, CBZ60, CBZ72, and CBZ75, also indicate a safer profile. Carcinogenicity represents one of the numerous toxicological endpoints associated with chemicals, which raises considerable concern due to its harmful effects on human health. As the landscape of chemical exposure and cancer epidemiology evolves, it is imperative that the assessment of carcinogenicity adapts accordingly. The carcinogenicity scores (ranging from 0 to 0.3) for certain analogues, including CBZ53, CBZ55, CBZ63, CBZ68, CBZ71, CBZ73, CBZ74, and CBZ78, indicate a safer profile, making them appropriate for minimizing carcinogenic risks. The NR-AR and NR-AR-LBD scores of analogues found within the range of 0–0.3. The toxicity parameter scores are presented in Table 3.

**TABLE 3:** Toxicity profile of CBZ analogues.

Compound no.	H-HT	DILI	hERG	Ames	ROA	Carc	NR-AR	NR-AR-LBD
CBZ01	0.64	0.61	0.376	0.77	0.86	0.72	0.12	0.00
CBZ02	0.75	0.34	0.587	0.58	0.90	0.52	0.18	0.01
CBZ03	0.66	0.86	0.421	0.96	0.93	0.43	0.00	0.00
CBZ04	0.72	0.71	0.336	0.59	0.80	0.39	0.02	0.00
CBZ05	0.71	0.45	0.557	0.67	0.86	0.44	0.15	0.00
CBZ06	0.76	0.87	0.376	0.81	0.75	0.60	0.02	0.00
CBZ07	0.70	0.88	0.128	0.83	0.70	0.78	0.03	0.00
CBZ08	0.74	0.88	0.657	0.84	0.71	0.78	0.02	0.00
CBZ09	0.78	0.86	0.215	0.83	0.72	0.76	0.05	0.00
CBZ10	0.80	0.90	0.196	0.77	0.72	0.72	0.09	0.00
CBZ11	0.69	0.82	0.499	0.86	0.81	0.62	0.00	0.00
CBZ12	0.79	0.67	0.428	0.65	0.73	0.51	0.15	0.01
CBZ13	0.74	0.51	0.355	0.58	0.79	0.44	0.14	0.00
CBZ14	0.70	0.60	0.288	0.68	0.83	0.43	0.01	0.00
CBZ15	0.60	0.73	0.134	0.82	0.79	0.55	0.01	0.00
CBZ16	0.79	0.47	0.316	0.84	0.86	0.63	0.00	0.00
CBZ17	0.68	0.73	0.134	0.83	0.85	0.64	0.02	0.00
CBZ18	0.74	0.60	0.316	0.59	0.72	0.50	0.09	0.01
CBZ19	0.68	0.71	0.208	0.74	0.81	0.55	0.05	0.00
CBZ 20	0.81	0.88	0.343	0.85	0.79	0.72	0.07	0.00
CBZ21	0.68	0.75	0.393	0.65	0.72	0.62	0.10	0.00
CBZ22	0.76	0.83	0.357	0.83	0.82	0.72	0.00	0.00
CBZ23	0.73	0.55	0.458	0.66	0.79	0.64	0.06	0.00
CBZ24	0.69	0.30	0.555	0.66	0.87	0.53	0.02	0.00
CBZ25	0.67	0.42	0.260	0.81	0.75	0.59	0.06	0.01
CBZ26	0.77	0.82	0.240	0.80	0.83	0.60	0.00	0.00
CBZ27	0.78	0.47	0.223	0.83	0.61	0.65	0.11	0.01
CBZ28	0.73	0.64	0.345	0.69	0.81	0.42	0.01	0.00
CBZ29	0.71	0.33	0.271	0.75	0.71	0.57	0.04	0.01
CBZ30	0.65	0.61	0.421	0.47	0.71	0.79	0.03	0.00
CBZ31	0.70	0.63	0.327	0.77	0.71	0.63	0.04	0.00
CBZ32	0.73	0.89	0.312	0.76	0.80	0.47	0.11	0.00
CBZ33	0.70	0.74	0.475	0.74	0.71	0.51	0.11	0.00
CBZ34	0.70	0.61	0.299	0.57	0.80	0.42	0.11	0.00
CBZ35	0.65	0.37	0.335	0.82	0.78	0.65	0.01	0.00
CBZ36	0.51	0.61	0.141	0.94	0.94	0.60	0.03	0.00
CBZ37	0.80	0.80	0.303	0.66	0.71	0.58	0.01	0.00
CBZ38	0.69	0.87	0.357	0.79	0.73	0.58	0.03	0.00
CBZ39	0.71	0.68	0.367	0.72	0.69	0.53	0.17	0.00
CBZ40	0.65	0.82	0.263	0.87	0.86	0.63	0.01	0.00
CBZ41	0.79	0.89	0.672	0.87	0.67	0.72	0.02	0.00
CBZ42	0.79	0.90	0.382	0.77	0.83	0.49	0.01	0.00
CBZ43	0.81	0.83	0.272	0.79	0.82	0.72	0.11	0.00
CBZ44	0.76	0.84	0.359	0.84	0.79	0.54	0.03	0.00
CBZ45	0.70	0.78	0.233	0.80	0.75	0.79	0.00	0.00
CBZ46	0.78	0.84	0.264	0.84	0.78	0.71	0.02	0.00
CBZ47	0.72	0.66	0.243	0.80	0.78	0.68	0.00	0.00
CBZ48	0.73	0.87	0.165	0.53	0.73	0.39	0.00	0.00
CBZ49	0.80	0.79	0.329	0.64	0.68	0.39	0.01	0.00
CBZ50	0.73	0.23	0.250	0.82	0.98	0.67	0.02	0.01
CBZ 51	0.70	0.87	0.169	0.76	0.80	0.68	0.20	0.03
CBZ52	0.92	0.96	0.609	0.88	0.46	0.57	0.69	0.06
CBZ53	0.95	0.96	0.421	0.93	0.27	0.12	0.23	0.10
CBZ54	0.80	0.94	0.691	0.87	0.68	0.48	0.52	0.06
CBZ55	0.89	1.00	0.482	0.01	0.13	0.22	0.55	0.06
CBZ56	0.91	0.98	0.867	0.26	0.19	0.76	0.33	0.05
CBZ57	0.84	0.98	0.277	0.11	0.27	0.74	0.04	0.01
CBZ58	0.84	0.94	0.796	0.89	0.25	0.42	0.70	0.14
CBZ59	0.94	0.98	0.430	0.62	0.40	0.88	0.69	0.13
CBZ60	0.92	0.98	0.742	0.34	0.29	0.86	0.55	0.47
CBZ61	0.89	0.98	0.525	0.40	0.75	0.74	0.45	0.15
CBZ62	0.91	0.96	0.530	0.90	0.67	0.89	0.44	0.12
CBZ63	0.30	0.97	0.007	0.68	0.75	0.27	0.33	0.18
CBZ64	0.32	0.96	0.182	0.86	0.43	0.40	0.24	0.18
CBZ65	0.32	0.96	0.182	0.86	0.43	0.40	0.24	0.18
CBZ66	0.52	0.96	0.326	0.92	0.60	0.56	0.62	0.15
CBZ67	0.93	0.81	0.798	0.87	0.64	0.43	0.01	0.00
CBZ68	0.80	0.93	0.337	0.81	0.48	0.06	0.01	0.00
CBZ69	0.78	0.96	0.106	0.76	0.31	0.35	0.43	0.07
CBZ70	0.80	0.94	0.691	0.87	0.68	0.48	0.52	0.06
CBZ71	0.95	0.96	0.421	0.93	0.27	0.12	0.23	0.10
CBZ72	0.93	0.96	0.245	0.93	0.22	0.67	0.04	0.05
CBZ73	0.91	0.97	0.457	0.93	0.45	0.06	0.23	0.07
CBZ74	0.84	0.94	0.730	0.91	0.70	0.27	0.58	0.03
CBZ75	0.76	0.98	0.608	0.85	0.25	0.57	0.72	0.03
CBZ76	0.97	0.93	0.836	0.87	0.61	0.07	0.04	0.01
CBZ77	0.93	0.95	0.561	0.86	0.56	0.66	0.67	0.06
CBZ78	0.86	0.95	0.641	0.80	0.39	0.21	0.64	0.08
CBZ79	0.82	0.94	0.436	0.60	0.48	0.73	0.66	0.39
CBZ80	0.93	0.89	0.421	0.57	0.54	0.78	0.63	0.13
CBZ81	0.90	0.97	0.305	0.21	0.53	0.85	0.70	0.14
CBZ	0.81	0.91	0.604	0.82	0.81	0.90	0.48	0.30

H-HT: human hepatotoxicity; DILI: drug induced liver injury; hERG: cardiotoxicity; Ames: mutagenicity; ROA: rat oral acute toxicity; Carc.: carcinogenicity; NR-AR: nuclear receptor-androgen receptor; NR-AR-LBD: nuclear receptor-androgen receptor-ligand binding domain.

### 3.7 Molecular docking analysis

Before the ligand’s docking study begins, we ensured the validity of the docking procedure by downloading a co-crystalized ligand receptor complex from the protein database (PDB ID: 3G5U). The structure of the co-crystallized ligand and the associated protein was prepared and saved in pdbqt format. Then we carried out a redocking experiment with the native ligand, under which Autodock Vina was used to transform the original ligand into the active site of the protein. We then evaluated the docked pose against the crystallographic pose by calculating the Root Mean Square Deviation (RMSD). The results showed that the RMSD value of the optimal pose (0.48 Å) is within the acceptable threshold of 2 Å.

**FIGURE 3:**
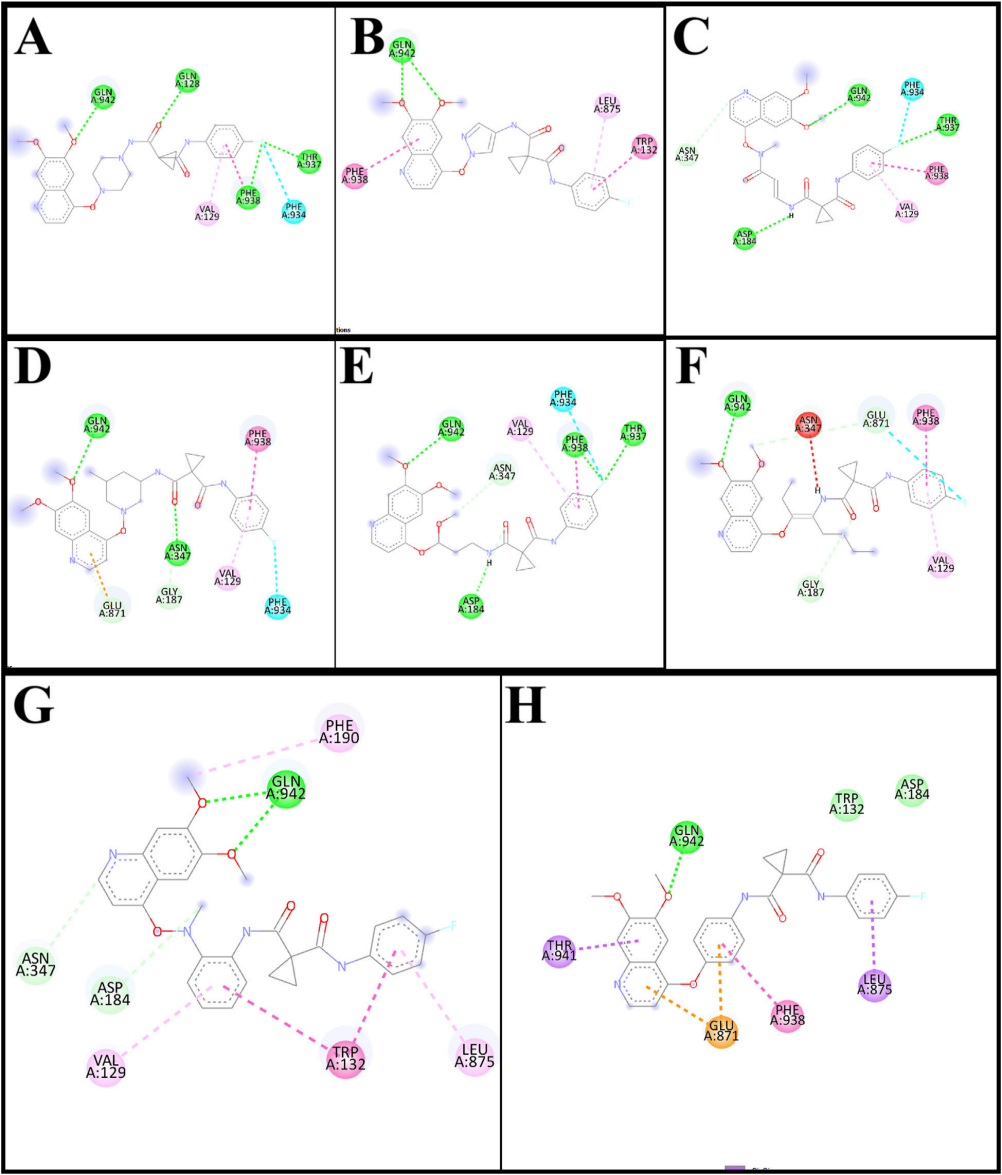
2D pose of ligands CBZ01 **(A)**, CBZ06 **(B)**, CBZ11 **(C)**, CBZ13 **(D)**, CBZ25 **(E)**, CBZ34 **(F)**, CBZ38 **(G)** and Cabozantinib **(H)**.

**FIGURE 4:**
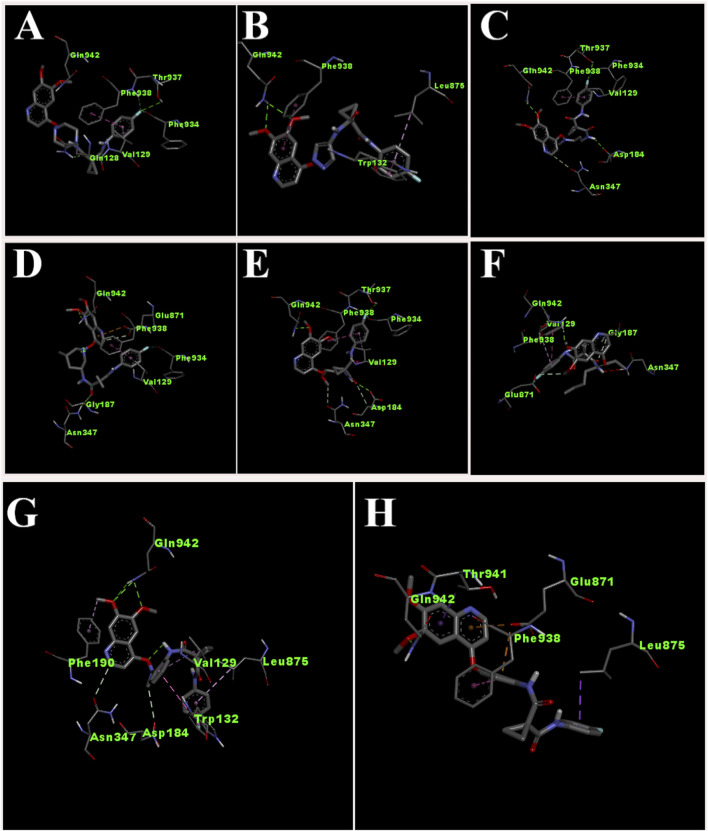
3D pose of ligands CBZ01 **(A)**, CBZ06 **(B)**, CBZ11 **(C)**, CBZ13 **(D)**, CBZ25 **(E)**, CBZ34 **(F)**, CBZ38 **(G)** and Cabozantinib **(H)**.

The interaction of designed ligands (Supplementary Table S1) and their docking score is being shown in Table 4, CBZ01 serves as a bioisostere of CBZ, characterized by the substitution of the phenyl group with a piperazine ring. The overall structure of CBZ01 closely resembles that of CBZ. Notably, the ligand CBZ01 exhibited the most favorable binding score of −8.0 kcal/mol. In the docked ADV complex, the residue GLN942 of the target protein forms hydrogen bonds with the oxygen of the methoxy group present in the quinoline ring of the ligand, with a distance of 1.9722 Å. The hydrogen bonds involving GLN942 are similar to those observed in CBZ. Additionally, residues GLN128 (2.5708 Å), PHE938 (2.9146 Å), and THR (2.9649 Å) demonstrated other hydrogen bond interactions with the carbonyl, phenyl, and fluorine atoms of the analogue. Furthermore, PHE934 established a halogenic bond with the fluorine atom of the fluorophenyl group of the ligand at a distance of 3.4667 Å. Figures 3A, 4A depict the 2D and 3D pose of the CBZ01, respectively.

**TABLE 4:** Docking score and interaction of the selected CBZ analogues.

Compound no.	Docking score (kcal/mol)	Interactions with distance	
		Hydrogen bonding	Other interactions
CBZ01	−8.0	GLN942 (1.97Å), GLN128 (2.57Å), PHE938 (2.91Å), THR937 (2.96Å)	PHE934 (3.47Å)
CBZ06	−7.8	GLN942 (2.47Å and 2.7Å)	PHE938 (5.53Å), TRP132 (7.31Å)
CBZ11	−7.7	GLN942 (2.57Å), THR937 (2.78Å)	PHE938 (4.72Å), PHE934 (3.42Å)
CBZ13	−8.4	GLN942 (2.13Å), ASN347 (2.46Å)	GLU871 (3.64Å), GLY187 (3.54Å), PHE938 (3.55Å), VAL129 (4.40Å)
CBZ25	−6.4	GLN942 (2.48Å), PHE938 (3Å), THR937 (2.71Å)	PHE934 (3.65Å)
CBZ34	−7.2	GLN942 (2.25Å)	GLY187 (3.57Å), GLU871 (3.16Å), PHE938 (4.42Å)
CBZ38	−8.0	GLN942 (2.71Å and 2.80Å)	TRP132 (4.83Å), PHE190 (5.43Å)
CBZ	−7.5	GLN942 (1.86Å)	GLU871 (3.60Å), LEU875 (3.70Å), THR941 (3.97Å), PHE938 (5.12Å)

CBZ06 serves as a bioisostere of CBZ, characterized by the substitution of the phenyl group with a pyrazole ring. The overall structure of CBZ06 closely resembles that of CBZ. Notably, the ligand CBZ06 exhibited the most advantageous binding score of −7.8 kcal/mol. In the docked ADV complex, the residue GLN942 of the target protein establishes hydrogen bonds with the oxygen atoms of the methoxy groups present in the quinoline ring, at distances of 2.4658 Å and 2.7057 Å. The hydrogen bonding interactions involving GLN942 are similar to those observed with CBZ. Additionally, residues PHE938 (5.5257 Å) and TRP132 (7.031 Å) demonstrate π-π stacking interactions with the quinoline and phenyl rings of CBZ, respectively. Figures 3B, 4B depict the 2D and 3D interactions of the ligand CBZ06, respectively.

CBZ11 serves as a bioisostere of CBZ, characterized by the substitution of the phenyl ring with a methyl amino-3-oxoprop-1-en-1-yl group. The overall structure of CBZ11 closely resembles that of CBZ. Notably, the ligand CBZ11 exhibited the most favorable binding score of −7.7 kcal/mol. In the docked ADV complex, the residue GLN942 of the target protein forms hydrogen bonds with the oxygen atoms of the methoxy groups present in the quinoline ring of the ligands, maintaining a distance of 2.5741 Å. The hydrogen bonds formed with GLN942 which was similar observed in the standard (CBZ). Additionally, residue THR937 demonstrates another hydrogen bond interaction with the amino and fluorine atoms of the analogue at a distance of 2.7844 Å. Furthermore, PHE934 forms a halogenic bond with the fluorine atom of the fluorophenyl group of the ligand, measured at a distance of 3.4229 Å PHE938 also engages in a π-π stacked interaction with the phenyl ring of the fluorophenyl group of the ligand, at a distance of 4.7230 Å. Figures 3C, 4C depict the 2D and 3D pose of the CBZ11, respectively.

CBZ13 serves as a bioisostere of CBZ, characterized by the substitution of the phenyl ring with a methylpiperidin-3-yl group. The overall structure of CBZ13 closely resembles that of CBZ. Notably, the ligand CBZ13 exhibited the most advantageous binding score of −8.4 kcal/mol. In the docked ADV complex, the residue GLN942 of the target protein engages in hydrogen bonding with the oxygen atoms of the methoxy groups located on the quinoline ring of the ligands, maintaining a distance of 2.1263 Å. Additionally, residue ASN347, at a distance of 2.4628 Å, displays another hydrogen bond interaction with the carbonyl group of the analogues. Residues GLU871 (3.6375 Å) and GLY187 (3.5368 Å) exhibit C-H bonding interactions with the quinoline ring and the carbonyl group, respectively. Furthermore, PHE938 and VAL129 establish π-π stacking and π-alkyl interactions with the fluorophenyl group of the ligand, at distances of 3.5515 Å and 4.3974 Å, respectively. Figures 3D, 4D depict the 2D and 3D pose of the CBZ13, respectively.

CBZ25 serves as a bioisostere of CBZ, characterized by the substitution of the phenyl ring with a methoxy propyl group. The overall structure of CBZ25 closely resembles that of CBZ. Notably, the ligand CBZ25 exhibited the most favorable binding score of −6.4 kcal/mol. In the docked ADV complex, the residue GLN942 of the target protein forms hydrogen bonds with the oxygen atoms of the methoxy groups present in the quinoline ring of the ligand, with a distance of 2.4776 Å. Additionally, residue PHE938 (2.9955 Å) and THR937 (2.7086 Å) demonstrate other hydrogen bond interactions with the fluorine atom of the CBZ analogue. Furthermore, PHE934 establishes a halogen bond with the fluorine atom of the fluorophenyl group of the ligand at a distance of 3.6510 Å. Figures 3E, 4E depict the 2D and 3D pose of the ligand CBZ25, respectively.

CBZ34 serves as a bioisostere of CBZ, characterized by the substitution of the phenyl ring with an oct-3-en-4-yl group. The overall structure of CBZ34 closely resembles that of CBZ. Notably, the ligand CBZ34 exhibited the most favorable binding score of −7.2 kcal/mol. In the docked ADV complex, the residue GLN942 of the target protein forms hydrogen bonds with the oxygen atoms of the methoxy groups located on the quinoline ring of the ligands, with a distance of 2.2471 Å. Additionally, residue GLY187 demonstrated a C-H bond with the carbonyl group of the analogues at a distance of 3.5685 Å. Furthermore, GLU871 established a halogen bond with the fluorine atom of the fluorophenyl group of the ligand, at a distance of 3.1584 Å. Lastly, residue PHE938 exhibited a π-π interaction with the phenyl ring of CBZ34 at a distance of 4.4169 Å. Figures 3F, 4F depict the 2D and 3D pose of the CBZ34, respectively.

CBZ38 serves as a bioisostere of CBZ, characterized by the substitution of the phenyl ring with a methyl amino phenyl group. The overall structure of CBZ38 closely resembles that of CBZ. The ligand CBZ38 exhibited the most favorable binding score of −8.0 kcal/mol. In the docked ADV complex, the residue GLN942 of the target protein forms hydrogen bonds with the oxygen atoms of the methoxy groups present in the quinoline ring of the ligand, at distances of 2.7084 Å and 2.7971 Å. Additionally, residue TRP132 demonstrates a π-π interaction at a distance of 4.8259 Å, while PHE190 forms a π-alkyl bond with the methoxy group of the quinoline in the ligand at a distance of 5.4208 Å. Figures 3G, 4G depict the 2D and 3D poseof the CBZ38, respectively.

The docking interaction of the standard drug (CBZ) exhibited a favorable binding score of −7.5 kcal/mol. The residue GLN942 of the target protein engages in hydrogen bonding with the oxygen atom of the methoxy group present in the quinoline ring, with a distance of 1.861 Å. The hydrogen bonds involving GLN942 are consistent across all ligands. Additionally, the residue GLU871 demonstrates a π-anionic interaction at a distance of 3.60 Å. The residue PHE190 forms a π-π interaction with the phenyl ring of quinoline in CBZ at a distance of 5.12 Å, while LEU875 shows a π-σ interaction. Figures 3H, 4H illustrate the 2D and 3D pose of the standard drug CBZ, respectively.

The comprehensive docking analysis showed that the designed ligands of CBZ, especially CBZ01 and CBZ13, had multiple interactions compared to CBZ itself. In the case of CBZ01, four hydrogen bonds were identified with residues GLN942, GLN128, PHE938, and THR937 of the target protein with distances of 1.97, 2.57, 2.91, and 2.96 Å, respectively. Furthermore, CBZ13 exhibited two hydrogen bonds with the target protein residues GLN942 and ASN347 of the target protein with distances of 2.13 and 2.46 Å, respectively. In contrast, CBZ showed a single interaction with residue GLN942 at a distance of 1.86 Å through hydrogen bonding. This could be the result of a change in the docking score between the parent compound cabozantinib (−7.5) and its analogues CBZ1 (−8.0) and CBZ13 (−8.4). The common amino acid residue GLN942 matches the key residue reported by Xiang et al. (2015). The authors suggested that residue GLN942 may play a crucial role in the reversal of P-gp-mediated MDR by CBZ analogues, which is achieved by inhibiting P-gp transporter function. Further validation of the docking study of these two ligands was carried out using a molecular dynamics (MD) simulation study. Examination of the MD simulation study revealed that the analogues CBZ01 and CBZ13 were found to be stable over the period of 100 ns.

### 3.8 Molecular dynamics (MD) simulation

MD simulations represent a computational approach that effectively models the physical behaviour of atoms and molecules, thereby facilitating advancements in drug discovery. The dynamic properties of molecular atoms were investigated through MD simulations. This approach encompasses a series of algorithms aimed at assessing and predicting the stability of protein-ligand complexes. It is acknowledged as a powerful independent method for precisely detecting alterations at both molecular and atomic levels. To comprehend the stability of protein-ligand complexes, it is crucial to investigate the interactions between ligand molecules and proteins. The assessment of a ligand’s stability and dynamic behaviour in relation to the protein relies heavily on MD simulations. We examined the MD simulation at 100 ns SPC water model-based simulation utilizing the simulation interaction diagram (SID). This examination yielded valuable insights into the deviations, fluctuations, and intermolecular interactions that transpired during the simulation. MD simulation was performed for the CBZ01, CBZ13, and CBZ38. Among these, the MD simulations for ligands CBZ01 and CBZ13 demonstrated stable complexes with the target protein, whereas the MD simulations for compound CBZ38 did not remain within the chosen parameters.

#### 3.8.1 RMSD and RMSF

**FIGURE 5:**
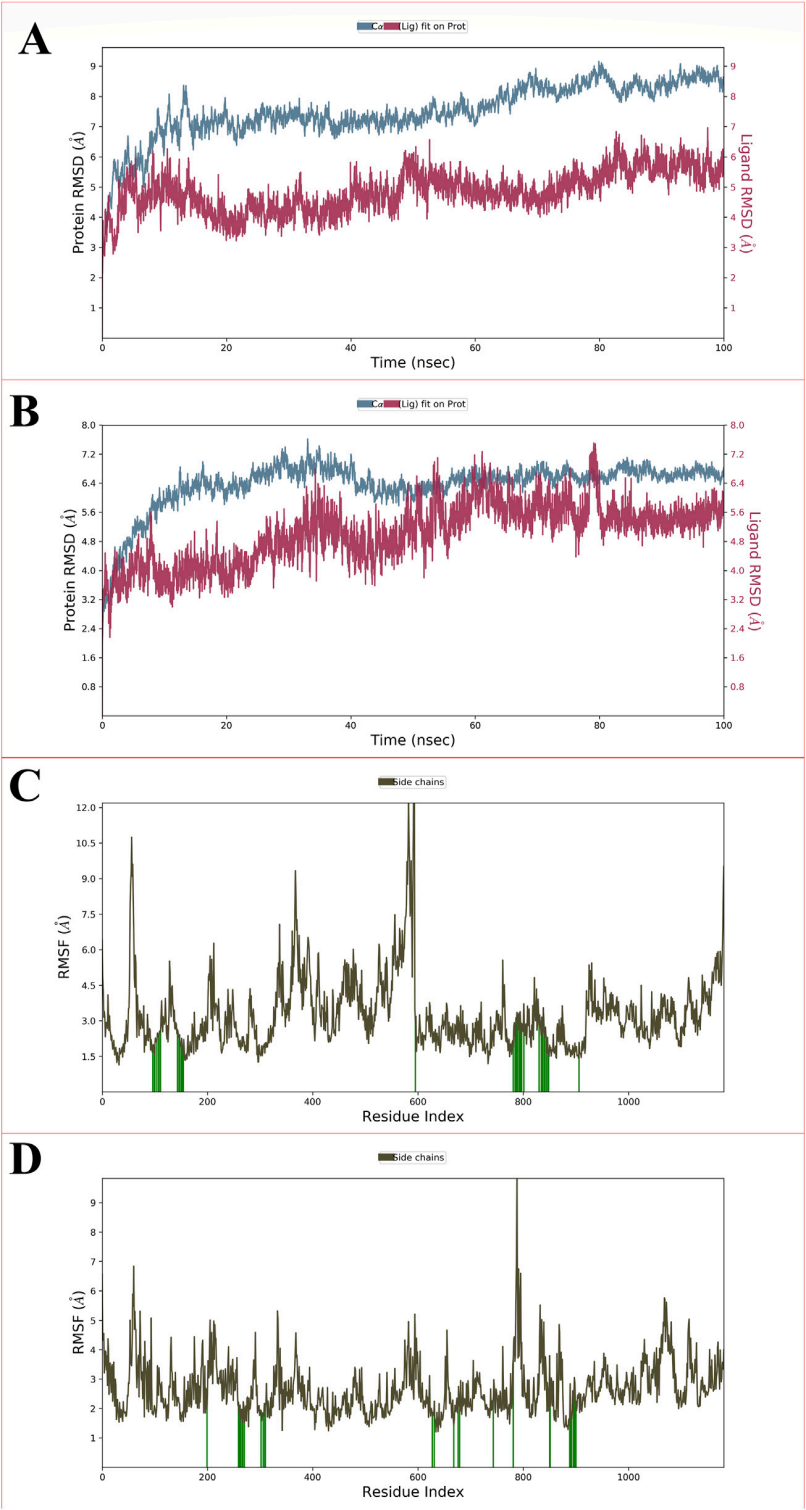
Showing the RMSD for P-glycoprotein with ligand CBZ01 **(A)** and ligand CBZ13 **(B)**; The protein-RMSF plot of P-glycoprotein concerning the CBZ01 **(C)** and ligand CBZ13 **(D)** throughout the 100 ns run.

The root mean square deviation (RMSD) quantifies the deviation of an atom’s molecule from its original structure or target over time. It is employed to evaluate the fluctuations in the protein’s backbone (C and N) during the 100 nsrun. Throughout the MD simulation, only minor variations in RMSD values related to the protein backbone were detected. For the protein complexed with the CBZ01, the ligand displayed a fluctuation of 2.5 Å, while the backbone RMSD initially ranged from 0 to 4.2 Å at the 0.50 ns? In contrast, when the protein was associated with the ligand CBZ13, the initial RMSD deviations for the ligand and protein were recorded at 2.8 Å and 2.9 Å, respectively. The P-gp complexed with CBZ01 showed an average RMSD of 1.57 Å, whereas the ligand exhibited an RMSD of 2.03 Å at the 100 ns? Conversely, the P-gp in complex with CBZ13 demonstrated an average RMSD of 1.61 Å, with the ligand showing an RMSD of 0.75 Å at the same time point. Initially, a lower deviation was observed from 0 to 25 ns, with slight fluctuations noted for two frames. Stable complexes (CBZ01) were identified from 25 to 50 ns, followed by a consistent deviation value with minimal changes from 50 to 100 ns? In the case of complex CBZ13, a lower deviation was also noted from 0 to 25 ns, with slight fluctuations observed for three frames. Stable complexes (CBZ01) were again identified from 25 to 50 ns and from 50 to 80 ns, followed by a consistent deviation value with minor changes from 80 to 100 ns? Overall, a stable RMSD value with slight deviations was observed from 80 to 100 ns, indicating that both complexes-maintained stability throughout the 100 ns simulation. The RMSD values for ligands CBZ01 and CBZ13 are illustrated in Figures 5A, B, respectively.

The root mean square fluctuation (RMSF) serves to measure the fluctuation of individual residues from their average fluctuation over a specified time period. The peaks identified in the protein-RMSF graph indicate the residues that display the most significant fluctuations throughout the simulation. Secondary structural components, such as alpha helices and beta strands, tend to exhibit less fluctuation than loop regions, owing to their enhanced rigidity relative to the unstructured portions of the protein. The green vertical bars on the graph denote protein residues that interact with the ligand. The variability among the other amino acid residues is considerably lower, reflecting a diminished level of fluctuation. During the 100 ns simulation, the ligands CBZ01 and CBZ13 showed minimal alterations in their interactions with each other, likely attributable to their effective engagement with various amino acid residues. The overall fluctuation observed is relatively low, providing valuable insights for future studies involving proteins with both ligands, CBZ01 and CBZ13. Additionally, the rigidity of the protein is reinforced by hydrogen bonds, pi-pi stacking, and the presence of secondary structural elements. In both scenarios illustrated in Figures 5C,D (CBZ01 and CBZ13), the fluctuations remain below 2 Å, indicating promising results.

#### 3.8.2 Intermolecular interactions

**FIGURE 6:**
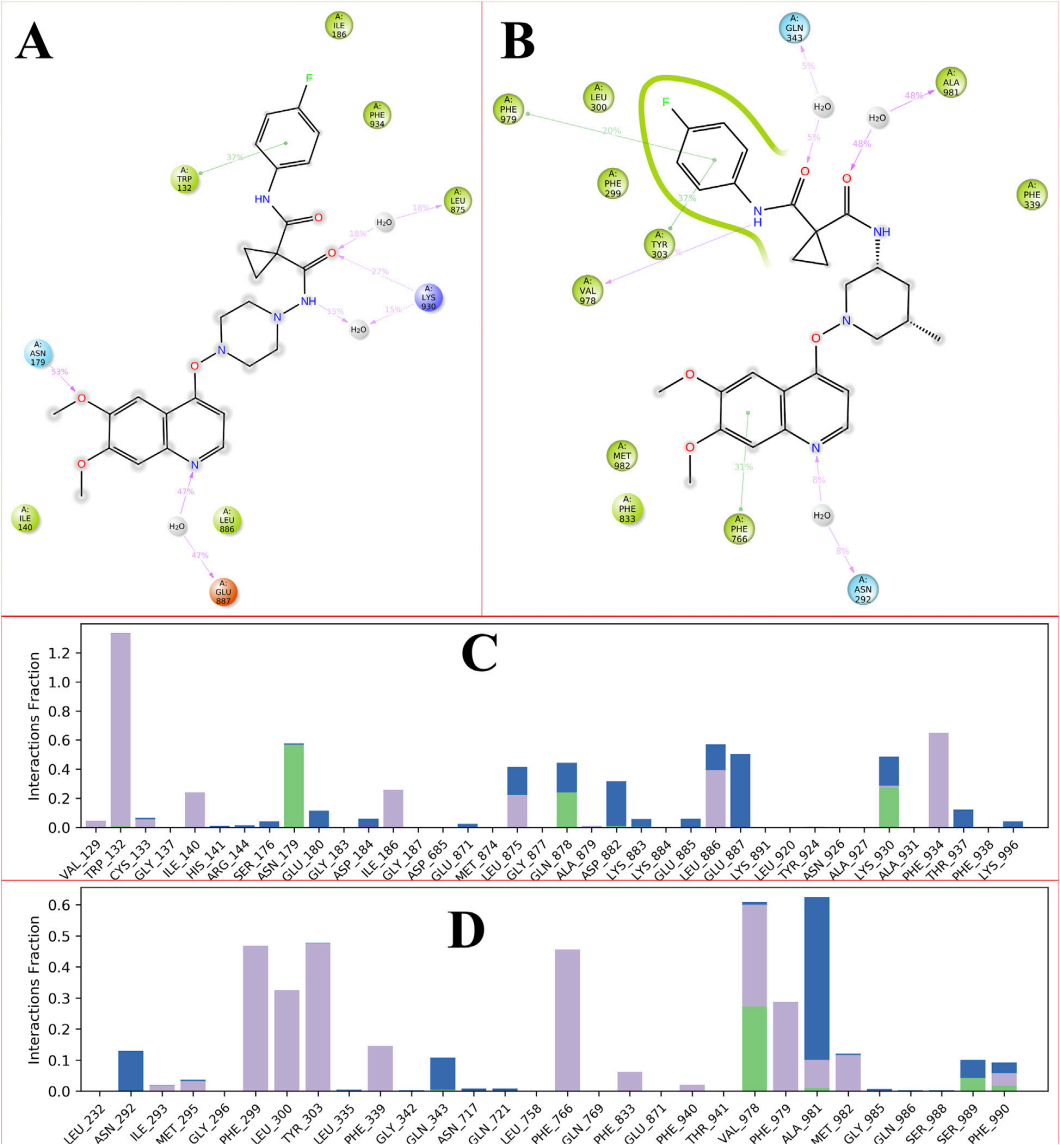
Showing the 2D-Summary of interacting atoms of P-glycoprotein with CBZ01 **(A)** and ligand CBZ13 **(B)**; The count of interactions in histogram form for P-glycoprotein with CBZ01 **(C)** and ligand CBZ13 **(D)** throughout the 100 ns run.

MD simulations facilitate the examination of intermolecular interactions, which are defined as the attractive or repulsive forces that exist between molecules. Over the course of a 100 ns simulation, we explored various binding interactions between the ligand and the protein. Numerous intramolecular interactions were identified in both complexes, encompassing hydrophobic, polar, water-mediated, and pi-pi stacking interactions. The presence of N, O, and NH atoms led to the formation of hydrophilic, hydrophobic, and cationic interactions across different percentiles. No direct interactions with carbon molecules were observed. Furthermore, the direction of the arrows indicates both donors and acceptors. The residue TRP132 engaged with the phenyl group through hydrophobic interactions. The carbonyl group of the amide was linked to LEU875 and LYS930 via a water bridge, exhibiting both hydrophobic and cationic characteristics. LYS930 also established a cationic interaction with the carbonyl group of the amide. The amino acid residue ASN179 interacted with the methoxy group of the ligands through polar interactions facilitated by a water bridge. Additionally, the nitrogen atom of the quinoline ring formed an anionic interaction with GLU887 through a water bridge. Figure 6A depicts the intramolecular interaction between the CBZ01 complex and the target protein.

The carbonyl group of the amide interacted with the GLN343 and ALA981 residues through polar and hydrophobic interactions, respectively. Moreover, the PHE979 and TYR303 residues formed hydrophobic interactions with the phenyl group and the carbonyl of the amide group, respectively. The NH of the amide and the phenyl group of the quinoline ring displayed hydrophobic interactions with the VAL292 and PHE766 residues, respectively. Additionally, the nitrogen atom of the quinoline ring established a hydrogen bond with the PHE766 residue, mediated by a water bridge. The intramolecular interactions of the CBZ13 complex with the target protein are depicted in Figure 6B. Figure 6C, D provide the histogram for ligands CBZ01 and CBZ13. The interactions between the protein and ligand are classified into four categories: ionic, hydrophobic, hydrogen bonding, and water bridges. The histogram illustrates that the stacked bar charts are standardized over the trajectory. The findings from the MD simulation indicate that both complexes, CBZ01 and CBZ13, demonstrated stability, characterized by lower RMDS and RMSF values, along with favorable interactions.

#### 3.8.3 Binding free energy calculations and residue decomposition

Numerous methods are available to assess the binding free energy of protein-ligand complexes. MM-PBSA and MM-GBSA are currently the leading techniques due to their effectiveness in predicting the interactions between small molecules and biological entities (Genheden and Ryde, 2015). Energies associated with the protein (PDB ID: 3G5U), along with those of the CBZ01-3G5U and CBZ13-3G5U complexes, as well as the entropy derived from these methodologies, were documented. An MM-GBSA analysis was carried out to determine whether variations in binding mode can be differentiated in the predicted binding-free energy between the two systems.

**FIGURE 7:**
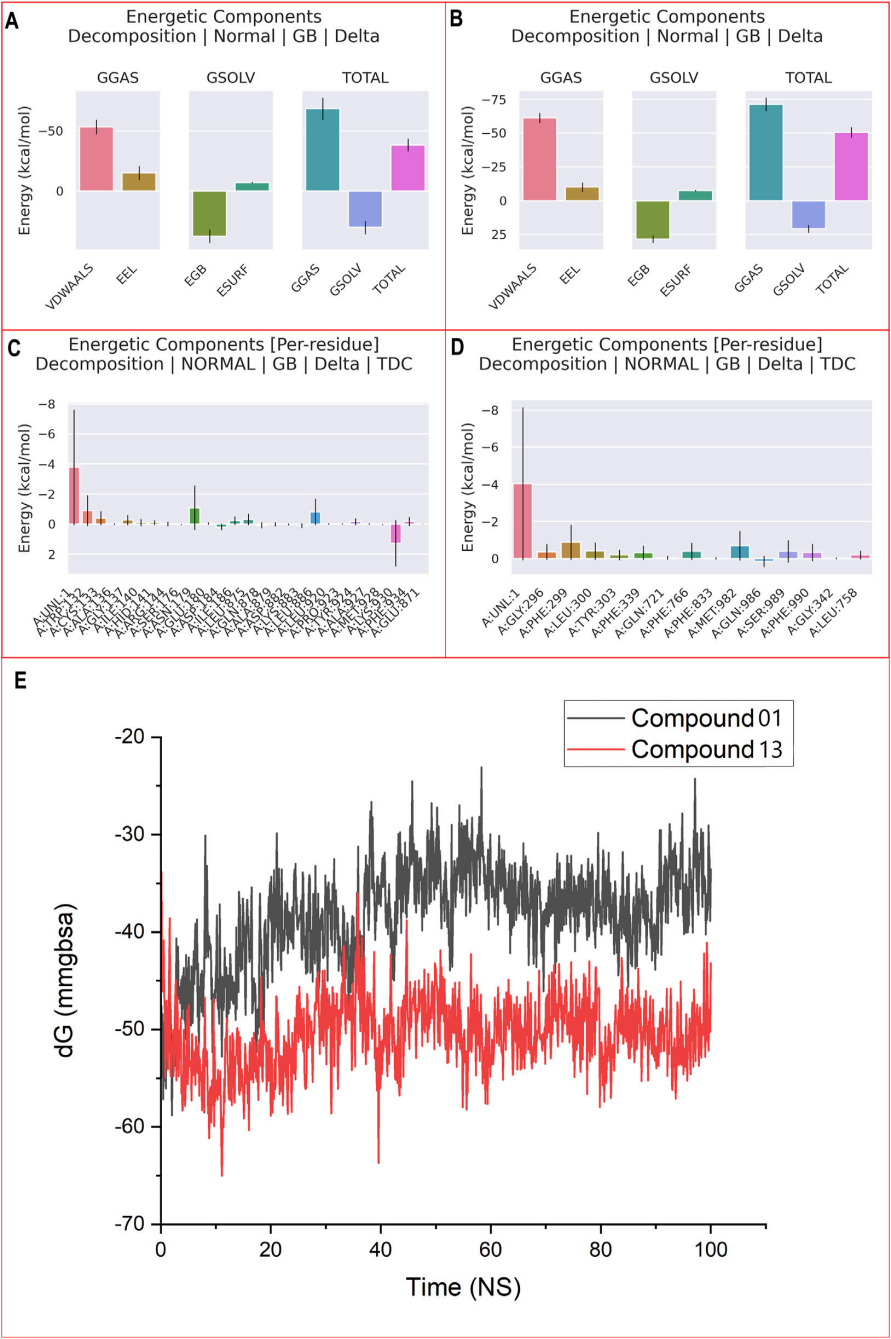
MM-PBSA binding free energy for ligand CBZ01-3G5U **(A)** and CBZ13-3G5U complexes **(B)**; per residue free energy decomposition for ligand CBZ01-3G5U **(C)** and CBZ13-3G5U complexes **(D)**; and MM-GBSA plot for CBZ01-3G5U and CBZ13-3G5U complexes **(E)**.

The study demonstrated that the total energetic component breakdown for the CBZ01-3G5U and CBZ13-3G5U complexes was −40 kcal/mol and −50 kcal/mol, respectively, as illustrated in Figures 7A, B. For the ligand CBZ01, an extensive analysis of the free-energy components revealed that ΔG_gas_ (−65 kcal/mol) was predominantly influenced within the protein environment. A minor decrease in ∆E_vdwalls_ (−55 kcal/mol) and a significant reduction in ∆E_EEl_ (−15 kcal/mol) contributed to the overall decline in ΔG_gas_ (−40 kcal/mol). In the case of ligand CBZ13, a thorough examination of the free-energy components indicated that ΔG_gas_ (−70 kcal/mol) was primarily affected within the protein. There was a slight decrease in ∆E_vdwalls_ (−60 kcal/mol) and a notable reduction in ∆E_EEl_ (−15 kcal/mol), leading to an overall decrease in ΔG_gas_ (−50 kcal/mol).

A detailed per-residue free energy decomposition analysis was performed on the CBZ01-3G5U and CBZ13-3G5U complexes to assess the contributions of various amino acid residues surrounding the binding site to the overall binding free energy (Figures 7C,D). The addition of 3G5U in the context of CBZ01 led to a reduction in energy contributions from several critical residues, particularly 136, 140, 141, 144, 176, 184, 186, 878, 879, 882, 883, 920, 923, 924, 927, 928, and 871. Similarly, the incorporation of 3G5U with respect to CBZ13 resulted in decreased energy contributions from key residues, namely, 296, 303, 721, 833, 342, and 758. The free energies of the CBZ01-3G5U and CBZ13-3G5U complexes were determined to be −38.40 and −50.56 kcal/mol, respectively, through the application of the GBSA solvation method (Figure 7E). We have computed the binding free energy for each five-frame interval over the course of the 100 ns simulation, totalling 5,000 frames (Supplementary Table S2). These results suggest that the CBZ13-3G5U complex shows considerable stability and a strong affinity for the P-gp receptor and thus effectively acts as an anti-cancer agent.

## 4 Conclusion

Certain cancers demonstrate varying degrees of resistance to medications, which significantly undermines the efficacy of chemotherapy in achieving favorable treatment outcomes. The cell membranes are characterized by the presence of P-gp, a crucial protein that expels several foreign substances from cells and may contribute to resistance to chemotherapeutic agents. The CBZ, a tyrosine kinase inhibitor, is utilized in the treatment of various types of cancer. P-gp plays a role in mediating multidrug resistance (MDR), a challenge that can be addressed by CBZ through the direct inhibition of its export mechanism. Consequently, P-gp represents a vital target for the development of anti-cancer therapeutics. Patients undergoing treatment with CBZ may experience a range of side effects, including liver dysfunction, hypertension, hand-foot syndrome, reduced appetite, and general malaise. To address these issues, modifications to the scaffold of the CBZ molecule are necessary to create safer, less toxic, and more effective agents against MDR. In this research, we developed novel CBZ analogues employing a bioisosteric approach. We assessed the pharmacokinetic and toxicological profiles of the newly designed CBZ bioisosteres using ADMETlab 3.0. Following the screening process, the selected ligands were docked against the target protein (PDB ID: 3G5U) utilizing ADV, and their interactions were examined using Discovery studio. The docking scores for the ligands ranged from −6.4 to −8.4 kcal/mol. The ligands CBZ01, CBZ06, CBZ11, CBZ13, CBZ25, CBZ34, and CBZ38 demonstrated favorable interactions with the target protein. The amino acid residue GLN942 was identified as a critical residue in the binding process, potentially contributing to the inhibitory activity of P-gp. We selected the top three ligands, CBZ01, CBZ13, and CBZ38, for a 100 ns MD simulation using the Schrödinger suite, based on their docking scores and interactions. The trajectory analysis indicated that CBZ01 and CBZ13 maintained stability when complexed with the target protein. This combined computational approach suggests that CBZ01 and CBZ13 may be promising candidates for further development of potential anticancer agents.

Further experimental studies are currently underway to substantiate the anti-cancer properties of the designed CBZ analogues.

## Data Availability

The original contributions presented in the study are included in the article/Supplementary Material, further inquiries can be directed to the corresponding author.
