# Supplementary material for: Designing novel cabozantinib analogues as p-glycoprotein inhibitors to target cancer cell resistance using molecular docking study, ADMET screening, bioisosteric approach, and molecular dynamics simulations
Source: Front Chem. 2025 Feb 27;13:1543075. doi: 10.3389/fchem.2025.1543075 (PMC11903459; doi:10.3389/fchem.2025.1543075)
Supplement: Supplementary file 1 [file Table1.docx]

**SUPPLEMENTARY FILE**

**Total Number of Supplementary File = 01**

**Supplementary Table 1. Structure and physicochemical properties of CBZ analogues.**

| **S. No.** | **Compound No.** | **Structure** | **MW** | **nHA** | **nHD** | **LogP** | **TPSA** |
| --- | --- | --- | --- | --- | --- | --- | --- |
| 1 | CBZ01 |  | 509.21 | 10 | 2 | 1.44 | 105.26 |
| 2 | CBZ02 |  | 508.21 | 9 | 2 | 2.27 | 102.02 |
| 3 | CBZ03 |  | 510.19 | 10 | 3 | 1.62 | 120.04 |
| 4 | CBZ04 |  | 523.21 | 9 | 2 | 3.14 | 115.85 |
| 5 | CBZ05 |  | 494.2 | 9 | 2 | 2.04 | 102.02 |
| 6 | CBZ06 |  | 491.16 | 10 | 2 | 2.56 | 116.6 |
| 7 | CBZ07 |  | 506.17 | 11 | 2 | 2.52 | 129.49 |
| 8 | CBZ08 |  | 507.17 | 12 | 4 | 1.95 | 155.51 |
| 9 | CBZ09 |  | 505.18 | 10 | 2 | 2.59 | 116.6 |
| 10 | CBZ10 |  | 505.18 | 10 | 2 | 2.34 | 116.6 |
| 11 | CBZ11 |  | 508.18 | 10 | 2 | 2.40 | 119.09 |
| 12 | CBZ12 |  | 493.2 | 8 | 2 | 2.99 | 98.78 |
| 13 | CBZ13 |  | 522.23 | 9 | 2 | 2.43 | 102.02 |
| 14 | CBZ14 |  | 496.21 | 9 | 2 | 2.16 | 102.02 |
| 15 | CBZ15 |  | 496.18 | 10 | 2 | 1.89 | 119.09 |
| 16 | CBZ16 |  | 497.21 | 10 | 4 | 1.66 | 128.04 |
| 17 | CBZ17 |  | 511.18 | 10 | 2 | 2.97 | 125.08 |
| 18 | CBZ18 |  | 510.23 | 9 | 4 | 2.22 | 124.8 |
| 19 | CBZ19 |  | 507.22 | 8 | 2 | 3.15 | 98.78 |
| 20 | CBZ 20 |  | 504.18 | 9 | 2 | 2.67 | 103.71 |
| 21 | CBZ21 |  | 507.22 | 8 | 2 | 3.71 | 98.78 |
| 22 | CBZ22 |  | 519.19 | 10 | 2 | 1.96 | 116.6 |
| 23 | CBZ23 |  | 522.23 | 9 | 2 | 2.46 | 102.02 |
| 24 | CBZ24 |  | 522.23 | 9 | 2 | 2.40 | 102.02 |
| 25 | CBZ25 |  | 497.2 | 9 | 2 | 2.36 | 108.01 |
| 26 | CBZ26 |  | 507.17 | 12 | 2 | 1.49 | 142.38 |
| 27 | CBZ27 |  | 497.2 | 9 | 3 | 1.86 | 119.01 |
| 28 | CBZ28 |  | 496.21 | 9 | 3 | 1.97 | 110.81 |
| 29 | CBZ29 |  | 495.22 | 8 | 2 | 3.02 | 98.78 |
| 30 | CBZ30 |  | 523.25 | 8 | 2 | 3.53 | 98.78 |
| 31 | CBZ31 |  | 497.2 | 9 | 2 | 2.29 | 108.01 |
| 32 | CBZ32 |  | 533.16 | 10 | 4 | 2.81 | 139.24 |
| 33 | CBZ33 |  | 531.18 | 9 | 3 | 2.68 | 119.01 |
| 34 | CBZ34 |  | 535.25 | 8 | 2 | 4.07 | 98.78 |
| 35 | CBZ35 |  | 496.21 | 9 | 2 | 1.98 | 102.02 |
| 36 | CBZ36 |  | 496.18 | 10 | 3 | 1.97 | 127.88 |
| 37 | CBZ37 |  | 538.22 | 10 | 2 | 2.80 | 119.09 |
| 38 | CBZ38 |  | 530.2 | 9 | 2 | 3.02 | 102.02 |
| 39 | CBZ39 |  | 531.18 | 9 | 3 | 2.56 | 119.01 |
| 40 | CBZ40 |  | 518.16 | 10 | 2 | 1.92 | 120.9 |
| 41 | CBZ41 |  | 515.19 | 8 | 2 | 3.10 | 98.78 |
| 42 | CBZ42 |  | 516.18 | 9 | 2 | 2.49 | 111.67 |
| 43 | CBZ43 |  | 518.2 | 9 | 2 | 3.04 | 103.71 |
| 44 | CBZ44 |  | 517.16 | 9 | 3 | 2.88 | 119.01 |
| 45 | CBZ45 |  | 534.2 | 11 | 2 | 1.78 | 119.84 |
| 46 | CBZ46 |  | 533.21 | 10 | 2 | 3.15 | 116.6 |
| 47 | CBZ47 |  | 522.17 | 12 | 2 | 2.00 | 138.72 |
| 48 | CBZ48 |  | 511.18 | 10 | 3 | 1.48 | 136.08 |
| 49 | CBZ49 |  | 523.14 | 9 | 2 | 3.08 | 108.01 |
| 50 | CBZ50 |  | 513.21 | 8 | 2 | 2.45 | 98.78 |
| 51 | CBZ 51 |  | 521.16 | 10 | 3 | 2.17 | 136.08 |
| 52 | CBZ52 |  | 489.17 | 8 | 2 | 4.26 | 98.78 |
| 53 | CBZ53 |  | 499.15 | 8 | 2 | 4.65 | 98.78 |
| 54 | CBZ54 |  | 490.17 | 9 | 4 | 3.14 | 124.8 |
| 55 | CBZ55 |  | 526.13 | 10 | 2 | 4.20 | 119.09 |
| 56 | CBZ56 |  | 498.17 | 8 | 1 | 4.69 | 87.5 |
| 57 | CBZ57 |  | 530.16 | 10 | 2 | 4.18 | 119.09 |
| 58 | CBZ58 |  | 515.19 | 8 | 1 | 4.98 | 89.99 |
| 59 | CBZ59 |  | 475.15 | 8 | 1 | 4.10 | 89.99 |
| 60 | CBZ60 |  | 563.19 | 8 | 1 | 5.34 | 89.99 |
| 61 | CBZ61 |  | 525.1 | 10 | 2 | 3.36 | 132.92 |
| 62 | CBZ62 |  | 503.15 | 9 | 2 | 4.59 | 111.31 |
| 63 | CBZ63 |  | 503.15 | 9 | 2 | 4.59 | 111.31 |
| 64 | CBZ64 |  | 630.98 | 8 | 2 | 5.46 | 98.78 |
| 65 | CBZ65 |  | 587.03 | 8 | 2 | 5.35 | 98.78 |
| 66 | CBZ66 |  | 587.03 | 8 | 2 | 5.35 | 98.78 |
| 67 | CBZ67 |  | 518.2 | 9 | 3 | 4.15 | 110.81 |
| 68 | CBZ68 |  | 517.19 | 9 | 2 | 2.55 | 98.78 |
| 69 | CBZ69 |  | 553.06 | 8 | 2 | 4.49 | 98.78 |
| 70 | CBZ70 |  | 490.17 | 9 | 4 | 3.14 | 124.8 |
| 71 | CBZ71 |  | 499.15 | 8 | 2 | 4.65 | 98.78 |
| 72 | CBZ72 |  | 506.16 | 10 | 4 | 3.11 | 134.53 |
| 73 | CBZ73 |  | 500.15 | 9 | 2 | 4.32 | 122.57 |
| 74 | CBZ74 |  | 504.18 | 9 | 4 | 2.62 | 124.8 |
| 75 | CBZ75 |  | 504.18 | 9 | 2 | 4.98 | 102.02 |
| 76 | CBZ76 |  | 504.18 | 9 | 3 | 3.97 | 110.81 |
| 77 | CBZ77 |  | 523.13 | 8 | 2 | 4.31 | 98.78 |
| 78 | CBZ78 |  | 505.16 | 9 | 2 | 3.72 | 108.01 |
| 79 | CBZ79 |  | 565.11 | 8 | 2 | 4.86 | 98.78 |
| 80 | CBZ80 |  | 528.18 | 9 | 2 | 4.49 | 122.57 |
| 81 | CBZ81 |  | 571.11 | 8 | 2 | 5.56 | 98.78 |
| STD | CBZ |  | 501.17 | 8 | 2 | 4.33 | 98.78 |

MW, Molecular weight; nHA, Number of hydrogen-bond acceptors; nHD, Number of hydrogen-bond donors; nRot, Number of rotatable bonds; TPSA, Topological polar surface area; log P, Logarithm of the n-octanol/water distribution coefficient.
