# Supplementary material for: Designing novel cabozantinib analogues as p-glycoprotein inhibitors to target cancer cell resistance using molecular docking study, ADMET screening, bioisosteric approach, and molecular dynamics simulations
Source: Front Chem. 2025 Feb 27;13:1543075. doi: 10.3389/fchem.2025.1543075 (PMC11903459; doi:10.3389/fchem.2025.1543075)
Supplement: Supplementary file 2 [file Table2.docx]

**Supplementary Table 2.** Binding free energy of CBZ01-3G5U and CBZ01-3G5U complexes.

| **Frames** | **Time (NS)** | **CBZ01** | **CBZ13** |
| --- | --- | --- | --- |
| 1 | 0.1 | -47.57 | -33.81 |
| 6 | 0.2 | -52.49 | -45.74 |
| 11 | 0.3 | -48.39 | -46.11 |
| 16 | 0.4 | -57.18 | -42.22 |
| 21 | 0.5 | -52.15 | -40.83 |
| 26 | 0.6 | -55.26 | -46.2 |
| 31 | 0.7 | -47.18 | -48.49 |
| 36 | 0.8 | -49.36 | -54.97 |
| 41 | 0.9 | -56.06 | -52.44 |
| 46 | 1 | -49.08 | -47.02 |
| 51 | 1.1 | -51.46 | -49.13 |
| 56 | 1.2 | -48.57 | -52.05 |
| 61 | 1.3 | -49.46 | -54.07 |
| 66 | 1.4 | -47.97 | -47.73 |
| 71 | 1.5 | -45.9 | -42.6 |
| 76 | 1.6 | -48.45 | -38.56 |
| 81 | 1.7 | -47.2 | -53.8 |
| 86 | 1.8 | -50.15 | -54.83 |
| 91 | 1.9 | -52.97 | -51.71 |
| 96 | 2 | -58.83 | -52.49 |
| 101 | 2.1 | -51.97 | -51.38 |
| 106 | 2.2 | -52.85 | -50.04 |
| 111 | 2.3 | -52.64 | -54.78 |
| 116 | 2.4 | -53.77 | -52.83 |
| 121 | 2.5 | -53.77 | -49.17 |
| 126 | 2.6 | -48.86 | -46.8 |
| 131 | 2.7 | -50.63 | -44.9 |
| 136 | 2.8 | -40.64 | -55.25 |
| 141 | 2.9 | -46.96 | -50.19 |
| 146 | 3 | -48.5 | -46.64 |
| 151 | 3.1 | -41.48 | -52.41 |
| 156 | 3.2 | -46.85 | -54.81 |
| 161 | 3.3 | -45.92 | -52.59 |
| 166 | 3.4 | -45.65 | -50.03 |
| 171 | 3.5 | -47.31 | -52.1 |
| 176 | 3.6 | -46.04 | -48.83 |
| 181 | 3.7 | -44.86 | -56.57 |
| 186 | 3.8 | -48.91 | -56.02 |
| 191 | 3.9 | -44.51 | -58.36 |
| 196 | 4 | -47.89 | -58.23 |
| 201 | 4.1 | -45.28 | -54.84 |
| 206 | 4.2 | -43.65 | -50.04 |
| 211 | 4.3 | -49.04 | -52.09 |
| 216 | 4.4 | -44.66 | -57.86 |
| 221 | 4.5 | -50.99 | -57.08 |
| 226 | 4.6 | -42.32 | -53.19 |
| 231 | 4.7 | -47.58 | -52.59 |
| 236 | 4.8 | -48.88 | -48 |
| 241 | 4.9 | -48.63 | -55.62 |
| 246 | 5 | -43.86 | -53.49 |
| 251 | 5.1 | -42.59 | -47.47 |
| 256 | 5.2 | -45.54 | -53.18 |
| 261 | 5.3 | -49.01 | -53.8 |
| 266 | 5.4 | -49.49 | -53.48 |
| 271 | 5.5 | -49.6 | -53.24 |
| 276 | 5.6 | -43.53 | -57.31 |
| 281 | 5.7 | -45.95 | -50.45 |
| 286 | 5.8 | -49.34 | -52.64 |
| 291 | 5.9 | -45.05 | -54.7 |
| 296 | 6 | -41.93 | -52.27 |
| 301 | 6.1 | -45.35 | -54.63 |
| 306 | 6.2 | -41.33 | -52.45 |
| 311 | 6.3 | -39.13 | -55.68 |
| 316 | 6.4 | -43.62 | -50.75 |
| 321 | 6.5 | -43.86 | -54.02 |
| 326 | 6.6 | -38.69 | -51.75 |
| 331 | 6.7 | -48.71 | -54.5 |
| 336 | 6.8 | -43.22 | -53.21 |
| 341 | 6.9 | -52.62 | -51.34 |
| 346 | 7 | -47.97 | -56.82 |
| 351 | 7.1 | -46.83 | -52.99 |
| 356 | 7.2 | -45.85 | -55.14 |
| 361 | 7.3 | -44.31 | -55.23 |
| 366 | 7.4 | -48.21 | -54.36 |
| 371 | 7.5 | -43.94 | -58.33 |
| 376 | 7.6 | -46.57 | -57.38 |
| 381 | 7.7 | -48.63 | -52.3 |
| 386 | 7.8 | -44.88 | -51.93 |
| 391 | 7.9 | -43.98 | -58.55 |
| 396 | 8 | -38.42 | -46.71 |
| 401 | 8.1 | -30.07 | -53.78 |
| 406 | 8.2 | -42.68 | -52.01 |
| 411 | 8.3 | -40.32 | -53.15 |
| 416 | 8.4 | -33.18 | -52.25 |
| 421 | 8.5 | -39.04 | -55.52 |
| 426 | 8.6 | -42.57 | -58.65 |
| 431 | 8.7 | -40.39 | -57.6 |
| 436 | 8.8 | -40.5 | -61.18 |
| 441 | 8.9 | -41.77 | -56.48 |
| 446 | 9 | -41.21 | -55.9 |
| 451 | 9.1 | -41.5 | -57.27 |
| 456 | 9.2 | -46.93 | -59.57 |
| 461 | 9.3 | -44.71 | -55.2 |
| 466 | 9.4 | -52.36 | -55.85 |
| 471 | 9.5 | -49.65 | -57.81 |
| 476 | 9.6 | -46.38 | -46.82 |
| 481 | 9.7 | -47.23 | -59.93 |
| 486 | 9.8 | -45.93 | -51.79 |
| 491 | 9.9 | -46.81 | -57.38 |
| 496 | 10 | -44.48 | -55.18 |
| 501 | 10.1 | -43.11 | -55.43 |
| 506 | 10.2 | -38.28 | -56.47 |
| 511 | 10.3 | -46.88 | -57.22 |
| 516 | 10.4 | -47.9 | -54.14 |
| 521 | 10.5 | -46.66 | -56.33 |
| 526 | 10.6 | -49.44 | -56.74 |
| 531 | 10.7 | -37.13 | -55.77 |
| 536 | 10.8 | -43.04 | -54.28 |
| 541 | 10.9 | -47.77 | -57.43 |
| 546 | 11 | -46.31 | -61.1 |
| 551 | 11.1 | -52.79 | -65.03 |
| 556 | 11.2 | -44.57 | -60.65 |
| 561 | 11.3 | -44.66 | -59.04 |
| 566 | 11.4 | -48.01 | -60.03 |
| 571 | 11.5 | -47.07 | -55.06 |
| 576 | 11.6 | -41.62 | -57.51 |
| 581 | 11.7 | -45.12 | -51.04 |
| 586 | 11.8 | -45.67 | -50.83 |
| 591 | 11.9 | -43.6 | -50.82 |
| 596 | 12 | -50.41 | -48.84 |
| 601 | 12.1 | -45.92 | -53.39 |
| 606 | 12.2 | -44.42 | -53.97 |
| 611 | 12.3 | -48.4 | -56.52 |
| 616 | 12.4 | -45.45 | -57.18 |
| 621 | 12.5 | -47.86 | -56.12 |
| 626 | 12.6 | -42.26 | -50.66 |
| 631 | 12.7 | -43.6 | -57.33 |
| 636 | 12.8 | -46.98 | -53.06 |
| 641 | 12.9 | -44.5 | -57.79 |
| 646 | 13 | -44.61 | -50.38 |
| 651 | 13.1 | -47.17 | -50.8 |
| 656 | 13.2 | -47.39 | -51.34 |
| 661 | 13.3 | -45.13 | -56.85 |
| 666 | 13.4 | -46.56 | -57.53 |
| 671 | 13.5 | -46.77 | -53.86 |
| 676 | 13.6 | -49.05 | -52.78 |
| 681 | 13.7 | -39.51 | -52.63 |
| 686 | 13.8 | -47.92 | -50.38 |
| 691 | 13.9 | -41.53 | -49.83 |
| 696 | 14 | -42.67 | -53.57 |
| 701 | 14.1 | -46.19 | -50.24 |
| 706 | 14.2 | -35.85 | -56.15 |
| 711 | 14.3 | -37.86 | -56.72 |
| 716 | 14.4 | -37.18 | -50.76 |
| 721 | 14.5 | -44.02 | -56.27 |
| 726 | 14.6 | -41.12 | -54.75 |
| 731 | 14.7 | -42.47 | -54.07 |
| 736 | 14.8 | -43.82 | -55.76 |
| 741 | 14.9 | -36.56 | -55.67 |
| 746 | 15 | -38.38 | -56.92 |
| 751 | 15.1 | -41.55 | -59.13 |
| 756 | 15.2 | -39.34 | -55.83 |
| 761 | 15.3 | -46.12 | -51.44 |
| 766 | 15.4 | -48.53 | -52.75 |
| 771 | 15.5 | -41.43 | -55.42 |
| 776 | 15.6 | -38 | -55.59 |
| 781 | 15.7 | -36.66 | -53.64 |
| 786 | 15.8 | -40.15 | -57.59 |
| 791 | 15.9 | -38.43 | -56.46 |
| 796 | 16 | -41.49 | -60.37 |
| 801 | 16.1 | -43.08 | -52.35 |
| 806 | 16.2 | -42.46 | -51.3 |
| 811 | 16.3 | -42.66 | -53.53 |
| 816 | 16.4 | -38.58 | -55.19 |
| 821 | 16.5 | -35.39 | -53.86 |
| 826 | 16.6 | -48.32 | -50.89 |
| 831 | 16.7 | -47.96 | -54.36 |
| 836 | 16.8 | -46.54 | -55.47 |
| 841 | 16.9 | -44.74 | -50.31 |
| 846 | 17 | -47.15 | -54.33 |
| 851 | 17.1 | -45.27 | -51.8 |
| 856 | 17.2 | -46.73 | -51.39 |
| 861 | 17.3 | -52.38 | -55.02 |
| 866 | 17.4 | -52.31 | -52.76 |
| 871 | 17.5 | -47.94 | -55.38 |
| 876 | 17.6 | -50.49 | -53.35 |
| 881 | 17.7 | -47.88 | -57.24 |
| 886 | 17.8 | -49.79 | -51.6 |
| 891 | 17.9 | -41.09 | -51.04 |
| 896 | 18 | -36.04 | -53.77 |
| 901 | 18.1 | -40.81 | -52.61 |
| 906 | 18.2 | -40.72 | -44.62 |
| 911 | 18.3 | -42.94 | -51.38 |
| 916 | 18.4 | -49.31 | -54.85 |
| 921 | 18.5 | -44.14 | -55.35 |
| 926 | 18.6 | -44.66 | -49.14 |
| 931 | 18.7 | -50.53 | -51.62 |
| 936 | 18.8 | -39.66 | -50.18 |
| 941 | 18.9 | -39.05 | -51.18 |
| 946 | 19 | -38.75 | -50.47 |
| 951 | 19.1 | -43.79 | -50.97 |
| 956 | 19.2 | -40.36 | -56.33 |
| 961 | 19.3 | -40.28 | -54.65 |
| 966 | 19.4 | -45.74 | -51.22 |
| 971 | 19.5 | -38.86 | -55.28 |
| 976 | 19.6 | -36.39 | -53.22 |
| 981 | 19.7 | -38.56 | -53.48 |
| 986 | 19.8 | -35.79 | -51.1 |
| 991 | 19.9 | -34.89 | -54.23 |
| 996 | 20 | -38.89 | -58.88 |
| 1001 | 20.1 | -34.3 | -53.36 |
| 1006 | 20.2 | -40.8 | -51.87 |
| 1011 | 20.3 | -45.15 | -58.55 |
| 1016 | 20.4 | -36.15 | -55.56 |
| 1021 | 20.5 | -39.99 | -55.44 |
| 1026 | 20.6 | -33.62 | -57.16 |
| 1031 | 20.7 | -41.05 | -53.08 |
| 1036 | 20.8 | -39.33 | -56.15 |
| 1041 | 20.9 | -39.54 | -55.47 |
| 1046 | 21 | -36.5 | -46.59 |
| 1051 | 21.1 | -29.85 | -46.89 |
| 1056 | 21.2 | -37.6 | -52.28 |
| 1061 | 21.3 | -42.94 | -48.28 |
| 1066 | 21.4 | -39.22 | -50.39 |
| 1071 | 21.5 | -33.47 | -52.91 |
| 1076 | 21.6 | -37.18 | -56.66 |
| 1081 | 21.7 | -37.71 | -53.26 |
| 1086 | 21.8 | -42.28 | -52.7 |
| 1091 | 21.9 | -43.93 | -56.33 |
| 1096 | 22 | -33.57 | -54.13 |
| 1101 | 22.1 | -39.03 | -52.8 |
| 1106 | 22.2 | -41.96 | -57.82 |
| 1111 | 22.3 | -41.83 | -55.12 |
| 1116 | 22.4 | -41.86 | -50.66 |
| 1121 | 22.5 | -40.67 | -52.19 |
| 1126 | 22.6 | -36.82 | -52.86 |
| 1131 | 22.7 | -39.23 | -54.89 |
| 1136 | 22.8 | -37.8 | -51.29 |
| 1141 | 22.9 | -40.33 | -52.58 |
| 1146 | 23 | -36 | -57.43 |
| 1151 | 23.1 | -42.18 | -58.7 |
| 1156 | 23.2 | -40.27 | -49.04 |
| 1161 | 23.3 | -37.92 | -51.54 |
| 1166 | 23.4 | -40.71 | -51.58 |
| 1171 | 23.5 | -38 | -53.52 |
| 1176 | 23.6 | -38.42 | -51.96 |
| 1181 | 23.7 | -40.94 | -55.9 |
| 1186 | 23.8 | -41.09 | -58.48 |
| 1191 | 23.9 | -37.45 | -50.35 |
| 1196 | 24 | -40.72 | -54.03 |
| 1201 | 24.1 | -41.71 | -53.94 |
| 1206 | 24.2 | -35.09 | -54.54 |
| 1211 | 24.3 | -33.6 | -51.66 |
| 1216 | 24.4 | -34.51 | -48.81 |
| 1221 | 24.5 | -32.97 | -55.15 |
| 1226 | 24.6 | -38.18 | -51.19 |
| 1231 | 24.7 | -36.46 | -47.92 |
| 1236 | 24.8 | -39.31 | -46.52 |
| 1241 | 24.9 | -39.76 | -48.08 |
| 1246 | 25 | -38.55 | -51.28 |
| 1251 | 25.1 | -39.06 | -48.1 |
| 1256 | 25.2 | -38.25 | -45.6 |
| 1261 | 25.3 | -38.25 | -47.78 |
| 1266 | 25.4 | -38.67 | -50.85 |
| 1271 | 25.5 | -36.76 | -50.28 |
| 1276 | 25.6 | -42.6 | -47.92 |
| 1281 | 25.7 | -42.86 | -51.14 |
| 1286 | 25.8 | -33.88 | -50.59 |
| 1291 | 25.9 | -34.49 | -52.29 |
| 1296 | 26 | -40.47 | -47.83 |
| 1301 | 26.1 | -41.65 | -54.14 |
| 1306 | 26.2 | -40.46 | -55.66 |
| 1311 | 26.3 | -41.9 | -56.11 |
| 1316 | 26.4 | -44.28 | -56.02 |
| 1321 | 26.5 | -38 | -54.09 |
| 1326 | 26.6 | -44.17 | -46.35 |
| 1331 | 26.7 | -43.97 | -47.11 |
| 1336 | 26.8 | -38.67 | -51.89 |
| 1341 | 26.9 | -35.16 | -49.09 |
| 1346 | 27 | -34.12 | -48.56 |
| 1351 | 27.1 | -34.59 | -49.25 |
| 1356 | 27.2 | -44.19 | -49.22 |
| 1361 | 27.3 | -43.51 | -51.57 |
| 1366 | 27.4 | -42.4 | -49.14 |
| 1371 | 27.5 | -39.78 | -52.77 |
| 1376 | 27.6 | -45.12 | -55.4 |
| 1381 | 27.7 | -36.08 | -50.62 |
| 1386 | 27.8 | -36.64 | -50.22 |
| 1391 | 27.9 | -40.54 | -50.44 |
| 1396 | 28 | -43.23 | -47.51 |
| 1401 | 28.1 | -33.2 | -47.21 |
| 1406 | 28.2 | -41.81 | -47.95 |
| 1411 | 28.3 | -37.63 | -44.73 |
| 1416 | 28.4 | -42.49 | -45.63 |
| 1421 | 28.5 | -44.69 | -47.26 |
| 1426 | 28.6 | -36.05 | -44.07 |
| 1431 | 28.7 | -45.93 | -48.52 |
| 1436 | 28.8 | -44.84 | -48.92 |
| 1441 | 28.9 | -42.49 | -52.53 |
| 1446 | 29 | -38.85 | -48.64 |
| 1451 | 29.1 | -38.26 | -48.88 |
| 1456 | 29.2 | -39.78 | -46.43 |
| 1461 | 29.3 | -37.59 | -51.12 |
| 1466 | 29.4 | -37.86 | -43.97 |
| 1471 | 29.5 | -42.67 | -48.13 |
| 1476 | 29.6 | -42.52 | -51.69 |
| 1481 | 29.7 | -41.74 | -47.89 |
| 1486 | 29.8 | -42.38 | -43.92 |
| 1491 | 29.9 | -34.15 | -49.76 |
| 1496 | 30 | -36.94 | -48.98 |
| 1501 | 30.1 | -37.44 | -53.6 |
| 1506 | 30.2 | -35.41 | -45.8 |
| 1511 | 30.3 | -45.02 | -49.46 |
| 1516 | 30.4 | -35.36 | -49.86 |
| 1521 | 30.5 | -34.88 | -48.8 |
| 1526 | 30.6 | -37.59 | -46.44 |
| 1531 | 30.7 | -38.16 | -49.49 |
| 1536 | 30.8 | -36.82 | -47.63 |
| 1541 | 30.9 | -40.78 | -55.46 |
| 1546 | 31 | -39.06 | -58.63 |
| 1551 | 31.1 | -41.36 | -52.4 |
| 1556 | 31.2 | -35.06 | -45.63 |
| 1561 | 31.3 | -36.93 | -48.91 |
| 1566 | 31.4 | -36.98 | -46.27 |
| 1571 | 31.5 | -32.48 | -53.78 |
| 1576 | 31.6 | -42.11 | -49.23 |
| 1581 | 31.7 | -38.92 | -47.53 |
| 1586 | 31.8 | -35.68 | -47.76 |
| 1591 | 31.9 | -37.96 | -48.62 |
| 1596 | 32 | -37.98 | -46.29 |
| 1601 | 32.1 | -40.79 | -52.77 |
| 1606 | 32.2 | -37.55 | -56.11 |
| 1611 | 32.3 | -43.98 | -50.81 |
| 1616 | 32.4 | -41.44 | -48.03 |
| 1621 | 32.5 | -40.47 | -51.58 |
| 1626 | 32.6 | -41.68 | -47.92 |
| 1631 | 32.7 | -42.17 | -49.66 |
| 1636 | 32.8 | -43.44 | -45.88 |
| 1641 | 32.9 | -42.98 | -45.24 |
| 1646 | 33 | -44.52 | -48.32 |
| 1651 | 33.1 | -37.32 | -45.64 |
| 1656 | 33.2 | -38.28 | -41.41 |
| 1661 | 33.3 | -37.99 | -41.51 |
| 1666 | 33.4 | -44.92 | -43.59 |
| 1671 | 33.5 | -40.94 | -49.21 |
| 1676 | 33.6 | -44.76 | -50.44 |
| 1681 | 33.7 | -42.09 | -46.44 |
| 1686 | 33.8 | -41.9 | -48.93 |
| 1691 | 33.9 | -39.08 | -46.49 |
| 1696 | 34 | -41.15 | -47.91 |
| 1701 | 34.1 | -47.84 | -48.95 |
| 1706 | 34.2 | -43.84 | -48.68 |
| 1711 | 34.3 | -46.06 | -49.07 |
| 1716 | 34.4 | -41.35 | -46.5 |
| 1721 | 34.5 | -46.21 | -48.23 |
| 1726 | 34.6 | -42.84 | -46.51 |
| 1731 | 34.7 | -39.81 | -54.19 |
| 1736 | 34.8 | -38.33 | -49.82 |
| 1741 | 34.9 | -45.36 | -45.24 |
| 1746 | 35 | -38.48 | -49.48 |
| 1751 | 35.1 | -41.1 | -47.83 |
| 1756 | 35.2 | -42.71 | -49.52 |
| 1761 | 35.3 | -41.31 | -53.81 |
| 1766 | 35.4 | -46.69 | -42.84 |
| 1771 | 35.5 | -40.48 | -41.93 |
| 1776 | 35.6 | -42.03 | -46.24 |
| 1781 | 35.7 | -35.94 | -43.13 |
| 1786 | 35.8 | -40.25 | -36.01 |
| 1791 | 35.9 | -31.22 | -36.01 |
| 1796 | 36 | -42.73 | -46.92 |
| 1801 | 36.1 | -40.78 | -41.33 |
| 1806 | 36.2 | -39.87 | -46.44 |
| 1811 | 36.3 | -45.03 | -50.17 |
| 1816 | 36.4 | -40.36 | -43.09 |
| 1821 | 36.5 | -45.54 | -48.07 |
| 1826 | 36.6 | -40.42 | -40.56 |
| 1831 | 36.7 | -43.43 | -52.38 |
| 1836 | 36.8 | -46.14 | -50.64 |
| 1841 | 36.9 | -44.1 | -46.07 |
| 1846 | 37 | -35.51 | -42.6 |
| 1851 | 37.1 | -33.97 | -49.5 |
| 1856 | 37.2 | -37.37 | -50.65 |
| 1861 | 37.3 | -32.11 | -53.56 |
| 1866 | 37.4 | -36.8 | -48.81 |
| 1871 | 37.5 | -33.1 | -47.24 |
| 1876 | 37.6 | -33.24 | -46.16 |
| 1881 | 37.7 | -37.72 | -52.13 |
| 1886 | 37.8 | -39.81 | -46.75 |
| 1891 | 37.9 | -37.59 | -47.98 |
| 1896 | 38 | -30.75 | -46.28 |
| 1901 | 38.1 | -27.58 | -48.55 |
| 1906 | 38.2 | -30.91 | -47.38 |
| 1911 | 38.3 | -26.64 | -48.45 |
| 1916 | 38.4 | -35.54 | -50.95 |
| 1921 | 38.5 | -28.18 | -50.27 |
| 1926 | 38.6 | -32.94 | -48.55 |
| 1931 | 38.7 | -36.02 | -46.87 |
| 1936 | 38.8 | -34.97 | -42.01 |
| 1941 | 38.9 | -38.18 | -52.97 |
| 1946 | 39 | -40.84 | -48.91 |
| 1951 | 39.1 | -36.94 | -54.25 |
| 1956 | 39.2 | -38.87 | -53.47 |
| 1961 | 39.3 | -34.89 | -56.28 |
| 1966 | 39.4 | -34.26 | -51.86 |
| 1971 | 39.5 | -34.33 | -53.4 |
| 1976 | 39.6 | -37.15 | -63.73 |
| 1981 | 39.7 | -36.92 | -49.58 |
| 1986 | 39.8 | -35.19 | -50.99 |
| 1991 | 39.9 | -35.4 | -56.05 |
| 1996 | 40 | -37.35 | -54.86 |
| 2001 | 40.1 | -33.89 | -51.21 |
| 2006 | 40.2 | -34.3 | -54.55 |
| 2011 | 40.3 | -32.39 | -53 |
| 2016 | 40.4 | -35.39 | -51.05 |
| 2021 | 40.5 | -32.15 | -52.3 |
| 2026 | 40.6 | -35.25 | -47.84 |
| 2031 | 40.7 | -31.84 | -51.51 |
| 2036 | 40.8 | -35.54 | -49.07 |
| 2041 | 40.9 | -38.85 | -51.81 |
| 2046 | 41 | -39.56 | -48.59 |
| 2051 | 41.1 | -37.31 | -52.9 |
| 2056 | 41.2 | -37.1 | -54.82 |
| 2061 | 41.3 | -40.24 | -48.42 |
| 2066 | 41.4 | -37.22 | -51.05 |
| 2071 | 41.5 | -35.07 | -53.57 |
| 2076 | 41.6 | -37.57 | -49.98 |
| 2081 | 41.7 | -37.55 | -48.05 |
| 2086 | 41.8 | -41.61 | -42.31 |
| 2091 | 41.9 | -38.69 | -46.32 |
| 2096 | 42 | -42.25 | -49.85 |
| 2101 | 42.1 | -42.53 | -52.3 |
| 2106 | 42.2 | -44.85 | -49.08 |
| 2111 | 42.3 | -39.99 | -48.75 |
| 2116 | 42.4 | -44.99 | -51.14 |
| 2121 | 42.5 | -41.53 | -51.48 |
| 2126 | 42.6 | -36.9 | -55.01 |
| 2131 | 42.7 | -39.58 | -55.17 |
| 2136 | 42.8 | -41.29 | -52.08 |
| 2141 | 42.9 | -32.14 | -51.65 |
| 2146 | 43 | -39.13 | -55.52 |
| 2151 | 43.1 | -35.15 | -49.47 |
| 2156 | 43.2 | -29.92 | -47.72 |
| 2161 | 43.3 | -31.6 | -55.65 |
| 2166 | 43.4 | -36.61 | -56.78 |
| 2171 | 43.5 | -29.74 | -50.74 |
| 2176 | 43.6 | -34.17 | -55.98 |
| 2181 | 43.7 | -40.11 | -47.75 |
| 2186 | 43.8 | -31.98 | -49.13 |
| 2191 | 43.9 | -35.93 | -49.04 |
| 2196 | 44 | -37.93 | -47.01 |
| 2201 | 44.1 | -33.23 | -54.87 |
| 2206 | 44.2 | -33.9 | -46.48 |
| 2211 | 44.3 | -36.07 | -49.43 |
| 2216 | 44.4 | -30.8 | -51.23 |
| 2221 | 44.5 | -38.09 | -46.48 |
| 2226 | 44.6 | -32.31 | -42.59 |
| 2231 | 44.7 | -38.52 | -38.79 |
| 2236 | 44.8 | -32.29 | -47.82 |
| 2241 | 44.9 | -37.02 | -49.03 |
| 2246 | 45 | -35.83 | -48.96 |
| 2251 | 45.1 | -34.74 | -50.68 |
| 2256 | 45.2 | -34.69 | -52.01 |
| 2261 | 45.3 | -29.34 | -45.12 |
| 2266 | 45.4 | -29.07 | -48.77 |
| 2271 | 45.5 | -29.39 | -49.81 |
| 2276 | 45.6 | -33.07 | -47.5 |
| 2281 | 45.7 | -24.51 | -47.24 |
| 2286 | 45.8 | -31.99 | -46.71 |
| 2291 | 45.9 | -32.7 | -47.13 |
| 2296 | 46 | -30.95 | -45.76 |
| 2301 | 46.1 | -37.12 | -48.11 |
| 2306 | 46.2 | -35.59 | -45.05 |
| 2311 | 46.3 | -31.65 | -48.13 |
| 2316 | 46.4 | -38.01 | -52.95 |
| 2321 | 46.5 | -34.26 | -47.92 |
| 2326 | 46.6 | -31.73 | -49.26 |
| 2331 | 46.7 | -35.98 | -48.53 |
| 2336 | 46.8 | -35.31 | -48.22 |
| 2341 | 46.9 | -37.11 | -44.95 |
| 2346 | 47 | -32.44 | -48.18 |
| 2351 | 47.1 | -34.71 | -46.46 |
| 2356 | 47.2 | -32.36 | -46.14 |
| 2361 | 47.3 | -32.57 | -47.08 |
| 2366 | 47.4 | -34.33 | -45.93 |
| 2371 | 47.5 | -39.88 | -51.69 |
| 2376 | 47.6 | -34.36 | -45.85 |
| 2381 | 47.7 | -34.65 | -47.48 |
| 2386 | 47.8 | -38.45 | -50 |
| 2391 | 47.9 | -34.36 | -43.87 |
| 2396 | 48 | -33.5 | -50.3 |
| 2401 | 48.1 | -37.55 | -46.49 |
| 2406 | 48.2 | -35.99 | -49.53 |
| 2411 | 48.3 | -38.13 | -49.6 |
| 2416 | 48.4 | -30.32 | -48.34 |
| 2421 | 48.5 | -29.55 | -51.31 |
| 2426 | 48.6 | -30.91 | -44.29 |
| 2431 | 48.7 | -31.28 | -49.98 |
| 2436 | 48.8 | -37.14 | -49.33 |
| 2441 | 48.9 | -33.07 | -49.41 |
| 2446 | 49 | -29.63 | -47.69 |
| 2451 | 49.1 | -36.91 | -48.87 |
| 2456 | 49.2 | -26.75 | -46.55 |
| 2461 | 49.3 | -32.44 | -45.09 |
| 2466 | 49.4 | -34.75 | -46.64 |
| 2471 | 49.5 | -29.68 | -48.86 |
| 2476 | 49.6 | -33.45 | -45.56 |
| 2481 | 49.7 | -36.66 | -52.03 |
| 2486 | 49.8 | -31.96 | -43.17 |
| 2491 | 49.9 | -33.82 | -43.49 |
| 2496 | 50 | -30.35 | -43.92 |
| 2501 | 50.1 | -35.59 | -45.54 |
| 2506 | 50.2 | -27.19 | -45.6 |
| 2511 | 50.3 | -34.24 | -47.47 |
| 2516 | 50.4 | -35.02 | -51.32 |
| 2521 | 50.5 | -29.14 | -47.39 |
| 2526 | 50.6 | -34.53 | -45.8 |
| 2531 | 50.7 | -31.65 | -51.66 |
| 2536 | 50.8 | -33.27 | -41.86 |
| 2541 | 50.9 | -36.88 | -45.85 |
| 2546 | 51 | -39.64 | -43.61 |
| 2551 | 51.1 | -36.7 | -48.45 |
| 2556 | 51.2 | -37.03 | -44.62 |
| 2561 | 51.3 | -36.47 | -44.58 |
| 2566 | 51.4 | -35.92 | -46.03 |
| 2571 | 51.5 | -34.71 | -49.2 |
| 2576 | 51.6 | -38.88 | -44.81 |
| 2581 | 51.7 | -34.31 | -50.68 |
| 2586 | 51.8 | -32.83 | -52.08 |
| 2591 | 51.9 | -35.3 | -51.57 |
| 2596 | 52 | -36.03 | -47.48 |
| 2601 | 52.1 | -39.49 | -53.44 |
| 2606 | 52.2 | -37.06 | -50.69 |
| 2611 | 52.3 | -36.78 | -53.38 |
| 2616 | 52.4 | -41.41 | -47.47 |
| 2621 | 52.5 | -39.83 | -47.61 |
| 2626 | 52.6 | -43.94 | -49.15 |
| 2631 | 52.7 | -41.2 | -51.1 |
| 2636 | 52.8 | -36.31 | -52.08 |
| 2641 | 52.9 | -28.98 | -47.66 |
| 2646 | 53 | -43.57 | -48.62 |
| 2651 | 53.1 | -39.41 | -45.78 |
| 2656 | 53.2 | -34.12 | -50.33 |
| 2661 | 53.3 | -34.76 | -48.8 |
| 2666 | 53.4 | -32.71 | -50.37 |
| 2671 | 53.5 | -35.52 | -45.2 |
| 2676 | 53.6 | -37.45 | -49.8 |
| 2681 | 53.7 | -33.41 | -50.4 |
| 2686 | 53.8 | -35.75 | -53.85 |
| 2691 | 53.9 | -32.44 | -49.65 |
| 2696 | 54 | -34.15 | -46.82 |
| 2701 | 54.1 | -32.62 | -51.31 |
| 2706 | 54.2 | -33.94 | -48.2 |
| 2711 | 54.3 | -26.98 | -45.13 |
| 2716 | 54.4 | -35.96 | -52.02 |
| 2721 | 54.5 | -33.46 | -47.96 |
| 2726 | 54.6 | -35.15 | -49.78 |
| 2731 | 54.7 | -28.82 | -52.33 |
| 2736 | 54.8 | -36.49 | -50.88 |
| 2741 | 54.9 | -35.78 | -58.01 |
| 2746 | 55 | -30.19 | -53.31 |
| 2751 | 55.1 | -29.08 | -50.58 |
| 2756 | 55.2 | -34.62 | -50.04 |
| 2761 | 55.3 | -31.69 | -53.36 |
| 2766 | 55.4 | -29.09 | -52.99 |
| 2771 | 55.5 | -38.33 | -52.21 |
| 2776 | 55.6 | -31.67 | -58.23 |
| 2781 | 55.7 | -28.5 | -52.75 |
| 2786 | 55.8 | -33.2 | -56.47 |
| 2791 | 55.9 | -33.93 | -47.75 |
| 2796 | 56 | -30.35 | -51.63 |
| 2801 | 56.1 | -31.01 | -52.2 |
| 2806 | 56.2 | -37.98 | -51.7 |
| 2811 | 56.3 | -37.3 | -50.11 |
| 2816 | 56.4 | -30.72 | -42.24 |
| 2821 | 56.5 | -30.49 | -52.31 |
| 2826 | 56.6 | -28.25 | -50.2 |
| 2831 | 56.7 | -32.46 | -49.64 |
| 2836 | 56.8 | -33.62 | -46.96 |
| 2841 | 56.9 | -35.23 | -50.36 |
| 2846 | 57 | -31.58 | -45.82 |
| 2851 | 57.1 | -33.67 | -48.74 |
| 2856 | 57.2 | -36.39 | -49.34 |
| 2861 | 57.3 | -31.79 | -54.74 |
| 2866 | 57.4 | -37.07 | -48.71 |
| 2871 | 57.5 | -31.31 | -45.39 |
| 2876 | 57.6 | -30.04 | -46.83 |
| 2881 | 57.7 | -32.48 | -51.62 |
| 2886 | 57.8 | -31 | -50.61 |
| 2891 | 57.9 | -28.17 | -48.24 |
| 2896 | 58 | -35.17 | -53.25 |
| 2901 | 58.1 | -30.58 | -49.85 |
| 2906 | 58.2 | -31.8 | -46.42 |
| 2911 | 58.3 | -23.09 | -51.62 |
| 2916 | 58.4 | -32.24 | -49.88 |
| 2921 | 58.5 | -30.74 | -55.56 |
| 2926 | 58.6 | -32.59 | -52.14 |
| 2931 | 58.7 | -32.24 | -52.95 |
| 2936 | 58.8 | -35.02 | -55.05 |
| 2941 | 58.9 | -33.07 | -51.79 |
| 2946 | 59 | -36.7 | -57.6 |
| 2951 | 59.1 | -35.6 | -48.46 |
| 2956 | 59.2 | -34.22 | -50.19 |
| 2961 | 59.3 | -36.57 | -52.19 |
| 2966 | 59.4 | -33.97 | -57.66 |
| 2971 | 59.5 | -31.53 | -50.15 |
| 2976 | 59.6 | -39.76 | -51.4 |
| 2981 | 59.7 | -33.85 | -49.23 |
| 2986 | 59.8 | -32.38 | -53.61 |
| 2991 | 59.9 | -29.93 | -54.87 |
| 2996 | 60 | -35.11 | -52.79 |
| 3001 | 60.1 | -34.85 | -53.66 |
| 3006 | 60.2 | -36.46 | -51.01 |
| 3011 | 60.3 | -33.69 | -51.13 |
| 3016 | 60.4 | -36.31 | -53.57 |
| 3021 | 60.5 | -35.29 | -52.85 |
| 3026 | 60.6 | -36.44 | -51.66 |
| 3031 | 60.7 | -38.42 | -46.71 |
| 3036 | 60.8 | -35.93 | -48.55 |
| 3041 | 60.9 | -36.43 | -48.84 |
| 3046 | 61 | -32.73 | -49.2 |
| 3051 | 61.1 | -33.99 | -51.4 |
| 3056 | 61.2 | -36.66 | -48.56 |
| 3061 | 61.3 | -39.26 | -45.68 |
| 3066 | 61.4 | -32.93 | -47.7 |
| 3071 | 61.5 | -30.11 | -51.66 |
| 3076 | 61.6 | -33.42 | -48.86 |
| 3081 | 61.7 | -34.89 | -48.07 |
| 3086 | 61.8 | -35.8 | -48.02 |
| 3091 | 61.9 | -35.22 | -52.15 |
| 3096 | 62 | -31.53 | -52.46 |
| 3101 | 62.1 | -35.16 | -52.02 |
| 3106 | 62.2 | -35.14 | -55.19 |
| 3111 | 62.3 | -35.52 | -50.42 |
| 3116 | 62.4 | -31.62 | -46.88 |
| 3121 | 62.5 | -37.09 | -51.18 |
| 3126 | 62.6 | -33.47 | -48.36 |
| 3131 | 62.7 | -35.04 | -52.09 |
| 3136 | 62.8 | -36.75 | -46.18 |
| 3141 | 62.9 | -37.45 | -46.61 |
| 3146 | 63 | -35.5 | -47.74 |
| 3151 | 63.1 | -38.02 | -49.6 |
| 3156 | 63.2 | -33.87 | -44.87 |
| 3161 | 63.3 | -34.4 | -49.98 |
| 3166 | 63.4 | -33.02 | -46.78 |
| 3171 | 63.5 | -42.2 | -49.08 |
| 3176 | 63.6 | -34.73 | -46.49 |
| 3181 | 63.7 | -40.31 | -47.3 |
| 3186 | 63.8 | -33.13 | -49.63 |
| 3191 | 63.9 | -39.55 | -52.51 |
| 3196 | 64 | -36.56 | -54.67 |
| 3201 | 64.1 | -34.63 | -52.48 |
| 3206 | 64.2 | -39.32 | -47.41 |
| 3211 | 64.3 | -32.91 | -48.1 |
| 3216 | 64.4 | -32.88 | -49.74 |
| 3221 | 64.5 | -35.01 | -51.34 |
| 3226 | 64.6 | -38.76 | -49.42 |
| 3231 | 64.7 | -37.04 | -46.63 |
| 3236 | 64.8 | -36.98 | -54.12 |
| 3241 | 64.9 | -36.94 | -49.57 |
| 3246 | 65 | -34.82 | -51.4 |
| 3251 | 65.1 | -38.08 | -47.84 |
| 3256 | 65.2 | -34.93 | -51.89 |
| 3261 | 65.3 | -34.48 | -47.42 |
| 3266 | 65.4 | -33.28 | -54.13 |
| 3271 | 65.5 | -35.52 | -49.56 |
| 3276 | 65.6 | -35.82 | -53.97 |
| 3281 | 65.7 | -38.14 | -50.56 |
| 3286 | 65.8 | -38.22 | -49.86 |
| 3291 | 65.9 | -30.88 | -52.22 |
| 3296 | 66 | -37.63 | -52.71 |
| 3301 | 66.1 | -37.82 | -54 |
| 3306 | 66.2 | -38.68 | -46.67 |
| 3311 | 66.3 | -32.19 | -52.46 |
| 3316 | 66.4 | -38.05 | -50.06 |
| 3321 | 66.5 | -32.97 | -50.07 |
| 3326 | 66.6 | -35.52 | -50.47 |
| 3331 | 66.7 | -36.89 | -45.12 |
| 3336 | 66.8 | -36.46 | -52.09 |
| 3341 | 66.9 | -43.42 | -47.28 |
| 3346 | 67 | -39.6 | -49.15 |
| 3351 | 67.1 | -39.19 | -50.77 |
| 3356 | 67.2 | -36.36 | -52.45 |
| 3361 | 67.3 | -33.36 | -46.25 |
| 3366 | 67.4 | -37.61 | -53.38 |
| 3371 | 67.5 | -36.67 | -54.03 |
| 3376 | 67.6 | -34.1 | -51.83 |
| 3381 | 67.7 | -39.79 | -52.11 |
| 3386 | 67.8 | -43.28 | -53.3 |
| 3391 | 67.9 | -40.25 | -50.73 |
| 3396 | 68 | -35.63 | -52.26 |
| 3401 | 68.1 | -34.64 | -47.14 |
| 3406 | 68.2 | -39.85 | -53.41 |
| 3411 | 68.3 | -43.61 | -51.37 |
| 3416 | 68.4 | -39.04 | -51.19 |
| 3421 | 68.5 | -33.75 | -50.09 |
| 3426 | 68.6 | -36.18 | -50.39 |
| 3431 | 68.7 | -35.85 | -50.04 |
| 3436 | 68.8 | -36.03 | -43.94 |
| 3441 | 68.9 | -34.82 | -50.96 |
| 3446 | 69 | -37.63 | -50.92 |
| 3451 | 69.1 | -36.89 | -51.1 |
| 3456 | 69.2 | -42.13 | -50.57 |
| 3461 | 69.3 | -38.59 | -51.85 |
| 3466 | 69.4 | -42.52 | -52.18 |
| 3471 | 69.5 | -37.9 | -50.42 |
| 3476 | 69.6 | -46.11 | -51.6 |
| 3481 | 69.7 | -40.7 | -50.63 |
| 3486 | 69.8 | -37.7 | -49.74 |
| 3491 | 69.9 | -38.53 | -53.53 |
| 3496 | 70 | -39.25 | -47.41 |
| 3501 | 70.1 | -37.01 | -46.7 |
| 3506 | 70.2 | -37.93 | -50.88 |
| 3511 | 70.3 | -39.34 | -46.41 |
| 3516 | 70.4 | -39.44 | -47.43 |
| 3521 | 70.5 | -35.35 | -50.34 |
| 3526 | 70.6 | -39.01 | -44.42 |
| 3531 | 70.7 | -34.71 | -49.97 |
| 3536 | 70.8 | -37.11 | -48.69 |
| 3541 | 70.9 | -38.34 | -52.96 |
| 3546 | 71 | -35.96 | -51.31 |
| 3551 | 71.1 | -38.09 | -44.11 |
| 3556 | 71.2 | -34.6 | -50.49 |
| 3561 | 71.3 | -36.52 | -48.8 |
| 3566 | 71.4 | -38.05 | -46.87 |
| 3571 | 71.5 | -37.75 | -43.38 |
| 3576 | 71.6 | -34.53 | -48.73 |
| 3581 | 71.7 | -44.24 | -44.83 |
| 3586 | 71.8 | -38.66 | -49.12 |
| 3591 | 71.9 | -43.04 | -48.36 |
| 3596 | 72 | -34.22 | -47.38 |
| 3601 | 72.1 | -31.93 | -49.86 |
| 3606 | 72.2 | -38.27 | -51.75 |
| 3611 | 72.3 | -41.12 | -47.76 |
| 3616 | 72.4 | -41.13 | -43.27 |
| 3621 | 72.5 | -32.91 | -46.14 |
| 3626 | 72.6 | -39.91 | -47.74 |
| 3631 | 72.7 | -39.75 | -45.7 |
| 3636 | 72.8 | -37.37 | -48.95 |
| 3641 | 72.9 | -35.61 | -46.34 |
| 3646 | 73 | -39.87 | -51.27 |
| 3651 | 73.1 | -36.18 | -49.75 |
| 3656 | 73.2 | -35.65 | -47.52 |
| 3661 | 73.3 | -39.28 | -49.34 |
| 3666 | 73.4 | -34.77 | -49.43 |
| 3671 | 73.5 | -37.92 | -45.63 |
| 3676 | 73.6 | -35.79 | -48.11 |
| 3681 | 73.7 | -39.2 | -44.95 |
| 3686 | 73.8 | -35.36 | -47.81 |
| 3691 | 73.9 | -32.14 | -50.5 |
| 3696 | 74 | -37.58 | -49.86 |
| 3701 | 74.1 | -33.71 | -50.02 |
| 3706 | 74.2 | -32.96 | -48.25 |
| 3711 | 74.3 | -36.9 | -51.33 |
| 3716 | 74.4 | -39.07 | -49.26 |
| 3721 | 74.5 | -40 | -47.86 |
| 3726 | 74.6 | -32.14 | -43.07 |
| 3731 | 74.7 | -37.03 | -50.51 |
| 3736 | 74.8 | -37.36 | -45.42 |
| 3741 | 74.9 | -36.18 | -47.83 |
| 3746 | 75 | -38.43 | -53.22 |
| 3751 | 75.1 | -34.26 | -48.75 |
| 3756 | 75.2 | -34.8 | -54.89 |
| 3761 | 75.3 | -41.99 | -52.2 |
| 3766 | 75.4 | -35.5 | -51.18 |
| 3771 | 75.5 | -35.24 | -44.86 |
| 3776 | 75.6 | -37.55 | -52.13 |
| 3781 | 75.7 | -37.88 | -50.31 |
| 3786 | 75.8 | -39.98 | -51.39 |
| 3791 | 75.9 | -34.3 | -47.69 |
| 3796 | 76 | -39.45 | -51.45 |
| 3801 | 76.1 | -34.06 | -46.33 |
| 3806 | 76.2 | -34.65 | -52.6 |
| 3811 | 76.3 | -38.07 | -54.37 |
| 3816 | 76.4 | -35.15 | -51.21 |
| 3821 | 76.5 | -33.11 | -48.13 |
| 3826 | 76.6 | -37.87 | -46.86 |
| 3831 | 76.7 | -37.03 | -53.09 |
| 3836 | 76.8 | -34.51 | -47.64 |
| 3841 | 76.9 | -38.47 | -44.3 |
| 3846 | 77 | -36.77 | -49.15 |
| 3851 | 77.1 | -37.5 | -45.84 |
| 3856 | 77.2 | -34.75 | -44.69 |
| 3861 | 77.3 | -37.83 | -48.79 |
| 3866 | 77.4 | -39.12 | -46.66 |
| 3871 | 77.5 | -40.83 | -56.66 |
| 3876 | 77.6 | -34.03 | -47.12 |
| 3881 | 77.7 | -32.46 | -46.68 |
| 3886 | 77.8 | -40.47 | -52.28 |
| 3891 | 77.9 | -40.06 | -43.01 |
| 3896 | 78 | -33.86 | -53.3 |
| 3901 | 78.1 | -36.95 | -50.54 |
| 3906 | 78.2 | -37.26 | -47.91 |
| 3911 | 78.3 | -34.18 | -48.64 |
| 3916 | 78.4 | -33.05 | -52.83 |
| 3921 | 78.5 | -33.84 | -49.48 |
| 3926 | 78.6 | -37.34 | -47.25 |
| 3931 | 78.7 | -39.91 | -47.84 |
| 3936 | 78.8 | -38.95 | -49.09 |
| 3941 | 78.9 | -40.4 | -52.59 |
| 3946 | 79 | -41.63 | -43.87 |
| 3951 | 79.1 | -34.7 | -43.59 |
| 3956 | 79.2 | -39.46 | -49.07 |
| 3961 | 79.3 | -35.73 | -45.84 |
| 3966 | 79.4 | -35.52 | -46.14 |
| 3971 | 79.5 | -29.8 | -46.26 |
| 3976 | 79.6 | -38.04 | -45.71 |
| 3981 | 79.7 | -38.05 | -46.06 |
| 3986 | 79.8 | -36.8 | -57.99 |
| 3991 | 79.9 | -39.49 | -53.38 |
| 3996 | 80 | -37.37 | -52.21 |
| 4001 | 80.1 | -40.27 | -51.3 |
| 4006 | 80.2 | -38.05 | -52.68 |
| 4011 | 80.3 | -35.04 | -55.75 |
| 4016 | 80.4 | -31.58 | -53.58 |
| 4021 | 80.5 | -32.5 | -52.76 |
| 4026 | 80.6 | -39.17 | -57.42 |
| 4031 | 80.7 | -35.87 | -53.33 |
| 4036 | 80.8 | -33.75 | -49.69 |
| 4041 | 80.9 | -39.22 | -55.52 |
| 4046 | 81 | -36.62 | -55.02 |
| 4051 | 81.1 | -36.32 | -51.92 |
| 4056 | 81.2 | -33.61 | -53.34 |
| 4061 | 81.3 | -37.38 | -51.08 |
| 4066 | 81.4 | -31.85 | -49.44 |
| 4071 | 81.5 | -38.61 | -50.03 |
| 4076 | 81.6 | -39.06 | -53.25 |
| 4081 | 81.7 | -34.95 | -50.26 |
| 4086 | 81.8 | -40.13 | -57.06 |
| 4091 | 81.9 | -37.19 | -54.93 |
| 4096 | 82 | -38.51 | -52.33 |
| 4101 | 82.1 | -38.19 | -51.56 |
| 4106 | 82.2 | -35.96 | -52.52 |
| 4111 | 82.3 | -38.77 | -52.35 |
| 4116 | 82.4 | -42.26 | -54.22 |
| 4121 | 82.5 | -38.43 | -49.29 |
| 4126 | 82.6 | -34.79 | -49.55 |
| 4131 | 82.7 | -35.69 | -47.48 |
| 4136 | 82.8 | -37.82 | -49.15 |
| 4141 | 82.9 | -42.13 | -49.28 |
| 4146 | 83 | -39.4 | -49.3 |
| 4151 | 83.1 | -39.23 | -52.4 |
| 4156 | 83.2 | -44.45 | -48.78 |
| 4161 | 83.3 | -43.34 | -48.01 |
| 4166 | 83.4 | -38.75 | -50.22 |
| 4171 | 83.5 | -38.62 | -45.96 |
| 4176 | 83.6 | -36.31 | -47.67 |
| 4181 | 83.7 | -37.81 | -50.49 |
| 4186 | 83.8 | -40.65 | -42.64 |
| 4191 | 83.9 | -36.4 | -43.72 |
| 4196 | 84 | -31.79 | -47.55 |
| 4201 | 84.1 | -36.78 | -51.47 |
| 4206 | 84.2 | -40.17 | -50.54 |
| 4211 | 84.3 | -39.48 | -50.79 |
| 4216 | 84.4 | -40.35 | -49.36 |
| 4221 | 84.5 | -36.32 | -46.43 |
| 4226 | 84.6 | -41.01 | -49.96 |
| 4231 | 84.7 | -40.93 | -51.25 |
| 4236 | 84.8 | -34.34 | -45.67 |
| 4241 | 84.9 | -43.47 | -46.88 |
| 4246 | 85 | -45.62 | -51.55 |
| 4251 | 85.1 | -34.05 | -52.96 |
| 4256 | 85.2 | -41.64 | -47.96 |
| 4261 | 85.3 | -38.22 | -52.97 |
| 4266 | 85.4 | -39.34 | -49.3 |
| 4271 | 85.5 | -43.28 | -47.51 |
| 4276 | 85.6 | -42.5 | -51.8 |
| 4281 | 85.7 | -43.53 | -45.94 |
| 4286 | 85.8 | -40.46 | -50.91 |
| 4291 | 85.9 | -37.23 | -47.31 |
| 4296 | 86 | -36.39 | -50.46 |
| 4301 | 86.1 | -40.21 | -53.37 |
| 4306 | 86.2 | -38.03 | -51.93 |
| 4311 | 86.3 | -43.74 | -46.89 |
| 4316 | 86.4 | -38.15 | -52.1 |
| 4321 | 86.5 | -33.92 | -47.15 |
| 4326 | 86.6 | -43.52 | -51.97 |
| 4331 | 86.7 | -37.65 | -50.04 |
| 4336 | 86.8 | -38.28 | -43.72 |
| 4341 | 86.9 | -35.57 | -46.63 |
| 4346 | 87 | -37.22 | -46.64 |
| 4351 | 87.1 | -41.06 | -50.31 |
| 4356 | 87.2 | -38.5 | -47.79 |
| 4361 | 87.3 | -36.78 | -49.76 |
| 4366 | 87.4 | -36.21 | -55.31 |
| 4371 | 87.5 | -33.19 | -52.19 |
| 4376 | 87.6 | -36.81 | -51.54 |
| 4381 | 87.7 | -38.56 | -49.69 |
| 4386 | 87.8 | -34.59 | -50.96 |
| 4391 | 87.9 | -37.23 | -46.81 |
| 4396 | 88 | -38.8 | -50.18 |
| 4401 | 88.1 | -39.24 | -51.63 |
| 4406 | 88.2 | -35.78 | -46.04 |
| 4411 | 88.3 | -42.31 | -49.72 |
| 4416 | 88.4 | -39.73 | -49.6 |
| 4421 | 88.5 | -36.19 | -49.66 |
| 4426 | 88.6 | -38.58 | -55.23 |
| 4431 | 88.7 | -38.73 | -49.18 |
| 4436 | 88.8 | -38.98 | -52.02 |
| 4441 | 88.9 | -38.68 | -47.7 |
| 4446 | 89 | -39.87 | -46.58 |
| 4451 | 89.1 | -40.63 | -50.98 |
| 4456 | 89.2 | -35.86 | -51.26 |
| 4461 | 89.3 | -42.93 | -52.53 |
| 4466 | 89.4 | -39.38 | -52.23 |
| 4471 | 89.5 | -40.61 | -46.95 |
| 4476 | 89.6 | -41.96 | -52.79 |
| 4481 | 89.7 | -39.16 | -49.79 |
| 4486 | 89.8 | -41.44 | -53.19 |
| 4491 | 89.9 | -43.89 | -52.8 |
| 4496 | 90 | -39.34 | -51.76 |
| 4501 | 90.1 | -41.77 | -54 |
| 4506 | 90.2 | -39.59 | -53.3 |
| 4511 | 90.3 | -35.44 | -52.5 |
| 4516 | 90.4 | -42.16 | -49.87 |
| 4521 | 90.5 | -36.73 | -53.74 |
| 4526 | 90.6 | -34.83 | -50.71 |
| 4531 | 90.7 | -29.54 | -49.47 |
| 4536 | 90.8 | -37.9 | -48.2 |
| 4541 | 90.9 | -29.52 | -52.33 |
| 4546 | 91 | -42.36 | -49.83 |
| 4551 | 91.1 | -38.39 | -49.46 |
| 4556 | 91.2 | -35.27 | -47.83 |
| 4561 | 91.3 | -37.82 | -48.6 |
| 4566 | 91.4 | -35.36 | -52.2 |
| 4571 | 91.5 | -33.13 | -52.17 |
| 4576 | 91.6 | -35.86 | -49.57 |
| 4581 | 91.7 | -32.22 | -49.69 |
| 4586 | 91.8 | -39.01 | -49.46 |
| 4591 | 91.9 | -32.55 | -48.49 |
| 4596 | 92 | -34.97 | -45.82 |
| 4601 | 92.1 | -34.8 | -54.79 |
| 4606 | 92.2 | -39.11 | -51.18 |
| 4611 | 92.3 | -32.85 | -51.29 |
| 4616 | 92.4 | -33.19 | -52.54 |
| 4621 | 92.5 | -28.9 | -49.14 |
| 4626 | 92.6 | -33.5 | -48.77 |
| 4631 | 92.7 | -29.69 | -45.79 |
| 4636 | 92.8 | -37.93 | -48.41 |
| 4641 | 92.9 | -31.68 | -55.76 |
| 4646 | 93 | -32.93 | -56.23 |
| 4651 | 93.1 | -31.63 | -54.66 |
| 4656 | 93.2 | -34.13 | -51.79 |
| 4661 | 93.3 | -31.42 | -52.34 |
| 4666 | 93.4 | -31.86 | -50.9 |
| 4671 | 93.5 | -30.88 | -54.76 |
| 4676 | 93.6 | -36.94 | -57.12 |
| 4681 | 93.7 | -32.17 | -54.59 |
| 4686 | 93.8 | -37.23 | -53.16 |
| 4691 | 93.9 | -33.84 | -47.21 |
| 4696 | 94 | -38.36 | -48.17 |
| 4701 | 94.1 | -33.92 | -53.68 |
| 4706 | 94.2 | -33.57 | -50.93 |
| 4711 | 94.3 | -33.88 | -53.34 |
| 4716 | 94.4 | -35.61 | -53.53 |
| 4721 | 94.5 | -30.79 | -45.06 |
| 4726 | 94.6 | -31.7 | -51.68 |
| 4731 | 94.7 | -33.49 | -53.68 |
| 4736 | 94.8 | -27.81 | -53.13 |
| 4741 | 94.9 | -38.29 | -50.54 |
| 4746 | 95 | -35.49 | -54.61 |
| 4751 | 95.1 | -40.23 | -53.42 |
| 4756 | 95.2 | -35.03 | -52.16 |
| 4761 | 95.3 | -35.38 | -51.89 |
| 4766 | 95.4 | -40.06 | -53.88 |
| 4771 | 95.5 | -34.07 | -48.42 |
| 4776 | 95.6 | -40.05 | -51.47 |
| 4781 | 95.7 | -35.37 | -55.26 |
| 4786 | 95.8 | -40.05 | -52.35 |
| 4791 | 95.9 | -38.69 | -50.56 |
| 4796 | 96 | -39.32 | -52.21 |
| 4801 | 96.1 | -31.42 | -48.88 |
| 4806 | 96.2 | -34.28 | -52.68 |
| 4811 | 96.3 | -33.55 | -54.62 |
| 4816 | 96.4 | -34.77 | -49.53 |
| 4821 | 96.5 | -37.63 | -49.2 |
| 4826 | 96.6 | -37.82 | -47.24 |
| 4831 | 96.7 | -40.06 | -50.35 |
| 4836 | 96.8 | -32.73 | -53.86 |
| 4841 | 96.9 | -29.53 | -45.87 |
| 4846 | 97 | -33.37 | -47.94 |
| 4851 | 97.1 | -24.26 | -50.07 |
| 4856 | 97.2 | -31.53 | -53.29 |
| 4861 | 97.3 | -30.81 | -48.34 |
| 4866 | 97.4 | -35.83 | -48.8 |
| 4871 | 97.5 | -29.86 | -47.43 |
| 4876 | 97.6 | -36.66 | -48.97 |
| 4881 | 97.7 | -38.23 | -51.17 |
| 4886 | 97.8 | -34.46 | -53.73 |
| 4891 | 97.9 | -31.93 | -49.43 |
| 4896 | 98 | -32.3 | -47.71 |
| 4901 | 98.1 | -32.58 | -52.63 |
| 4906 | 98.2 | -29.4 | -50.91 |
| 4911 | 98.3 | -32.97 | -52.91 |
| 4916 | 98.4 | -34.65 | -49.44 |
| 4921 | 98.5 | -37.43 | -49.99 |
| 4926 | 98.6 | -29.77 | -50.59 |
| 4931 | 98.7 | -36.02 | -48.93 |
| 4936 | 98.8 | -34.24 | -42.18 |
| 4941 | 98.9 | -34.4 | -48.76 |
| 4946 | 99 | -34.77 | -52.82 |
| 4951 | 99.1 | -40.27 | -49.1 |
| 4956 | 99.2 | -36.08 | -51.2 |
| 4961 | 99.3 | -38.81 | -41.1 |
| 4966 | 99.4 | -37.87 | -45.36 |
| 4971 | 99.5 | -37.73 | -49.36 |
| 4976 | 99.6 | -29.04 | -52.18 |
| 4981 | 99.7 | -31.77 | -47.92 |
| 4986 | 99.8 | -32.64 | -48.53 |
| 4991 | 99.9 | -38.87 | -50.28 |
| 4996 | 100 | -33.52 | -43.19 |
